# Impact of Ferric and Ferrous Iron on the Crystallization of Rare Earth Sulphate Hydrates

**DOI:** 10.1002/cssc.202500285

**Published:** 2025-07-17

**Authors:** Nitin Pawar, Alexandre Chagnes, Marie Christine Boiron, Michel Cathelineau, Michael Svärd, Kerstin Forsberg

**Affiliations:** ^1^ Department of Chemical Engineering KTH Royal Institute of Technology SE‐11428 Stockholm Sweden; ^2^ Université de Lorraine CNRS GeoRessources 54000 Nancy France

**Keywords:** antisolvent crystallization, impurity incorporation, purity, rare earth elements, recycling

## Abstract

Rare earth elements (REEs) are important for permanent magnets used in for example wind turbines and motors. There is an imbalance in supply and demand of this commodity and the REE have been identified as critical raw materials by the European Union. This study focuses on recovery of REEs from sulfuric acid solutions using antisolvent crystallization in recycling of magnet waste. Ethanol is used as an antisolvent to crystallize Nd_2_(SO_4_)_3_·8H_2_O and (Nd/Dy)_2_(SO_4_)_3_·8H_2_O. The impact of the presence of Fe in ferrous and ferric states, and of different seeding strategies, on the quality of the crystal product in terms of purity, crystal size, morphology and agglomeration has been investigated. Higher purity (above 99%) is obtained for seeded experiments and the purity is higher for higher seed loading and lower antisolvent dosing rate. Furthermore, Fe(III) has a higher tendency to be incorporated into the pure Nd phase compared to the Nd phase containing 10% of Dy, while Fe(II) is not detected in any of the phases. By balancing the addition of antisolvent and seed loading the optimum conditions in terms of high purity and productivity can be found. The results provide insights to improve the recovery of REEs as a pure concentrate.

## Graphical abstract

The study investigates recovery of rare earth elements from magnet waste by antisolvent crystallization by addition of ethanol. The impact of ferric and ferrous iron on crystal purity and morphology are examined. Fe(III) hinders crystal growth and incorporates into Nd_2_(SO_4_)_3_·8H_2_O but not the mixed Nd/Dy phase. Fe(II) is only precipitated as a separate phase.

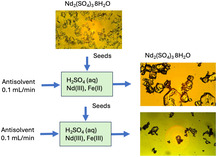

## 1 Introduction

Rare earth elements (REE) are recognized as the building blocks of many modern‐day technologies^[^ ^1^ ^]^ due to their critical role in hi‐tech applications such as motors of electric vehicles, wind turbine generators, and various consumer electronic appliances.^[^ ^2^ ^]^ The strongest permanent magnet type, NdFeB, contains 25–30 wt% of neodymium and 60–70% of iron.^[^ ^3^, ^4^ ^]^ The market for NdFeB magnets is growing at a rate of around 9.5% per year^[^ ^5^ ^]^ representing an increase from 34.5 to 54.1 billion USD for year 2021–2026. The global economy is heavily dependent on the need for these elements which are supplied mainly from mining.^[^ ^6^ ^]^

Over the last decade, REE recovery from spent magnets and waste streams has been a prioritized task in research and development globally. Due to the high demand and increased consumption, REEs are also at risk of supply chain disruptions.^[^ ^7^ ^]^ Therefore, it is important to support the primary supply in the future by reducing the burden of mining and to promote a circular economy.^[^ ^8^, ^9^ ^]^

The literature reports several processes for REE recovery from Nd‐based magnets based on hydrometallurgy and pyrometallurgy.^[^ ^2^, ^3^, ^4^, ^5^, ^7^, ^10^, ^11^, ^12^ ^]^ The hydrometallurgical methods show good potential in terms of not only recovery but also versatility and robustness. However, there are still challenges connected to low efficiency, poor selectivity (purity) of REEs, and high chemical waste generation.^[^ ^11^, ^12^ ^]^ Crystallization and precipitation can be applied to recover the REE as a mixed concentrate from impure leach solutions. The concentrate can then be further processed to recover the REE as for example, pure oxides or fluorides for new magnet production or to be used in other applications. The process can be designed to optimize key product properties such as crystal size, shape, polymorphic form, and purity by controlling the supersaturation, which is the driving force for crystallization.^[^ ^13^, ^14^ ^]^ Supersaturation can be generated by evaporation, temperature change, addition of another solvent known as antisolvent, or by reactive crystallization. Evaporative and cooling crystallization can be energy‐intensive processes dependent on heat transfer rate between solutions, which could introduce specific challenges in terms of scaling and product quality.^[^ ^15^ ^]^ Reaction crystallization can be applied to precipitate metal salts by adding a precipitation agent. It is generally difficult to control product properties such as purity in precipitation processes, and it can lead to large quantities of solid waste.^[^ ^16^ ^]^ Antisolvent crystallization is widely used in the pharmaceutical industry. Addition of another solvent can significantly alter the solubility of a solute in the mixed solvent system, which leads to generation of supersaturation.^[^ ^15^, ^17^, ^18^ ^]^ The antisolvent should have good miscibility with the initial solvent. Furthermore, in the case of crystallization of metal salts, a low dielectric constant of the antisolvent is favorable as it indicates a poor ability to dissolve ions.^[^ ^19^ ^]^

Only a few studies on the application of antisolvent crystallization in hydrometallurgy for the recovery of rare earth salts are reported in the literature.^[^ ^20^, ^21^, ^22^, ^23^, ^24^ ^]^ Antisolvent crystallization can be an attractive alternative for recovery of REE due to the relatively high mass fraction solubilities coupled with rather low molar solubilities.^[^ ^25^ ^]^ The REE sulphate salts obtained by antisolvent crystallization have the benefit of exhibiting a high aqueous solubility, facilitating easy redissolution and further purification and separation stages.^[^ ^23^ ^]^

During crystallization, impurities can be incorporated into crystal products by different mechanisms. Solution adhering to the surface and being trapped between crystals and separate precipitates can be counteracted by washing and centrifugation and possibly reslurrying in downstream processing. Lattice incorporation can be either kinetically or thermodynamically controlled.^[^ ^26^ ^]^ Nonequilibrium lattice impurity incorporation can be influenced by modifying the crystallization conditions or downstream processes.^[^ ^27^, ^28^ ^]^ In general, slow growth rate enhances purity but decreases productivity. Effective mixing generally enhances purity.^[^ ^29^ ^]^ The product crystal size distribution (CSD) and crystal shape have an impact on filtration and washing and thus an impact on product purity and energy consumption.^[^ ^30^ ^]^ Gaining an in‐depth understanding of the dominating mechanisms behind impurity incorporation can help in better design of processes to recover high‐purity products.

Improving local as well as bulk supersaturation control via controlled addition of antisolvent can be a strategy for promoting impurity rejection. The purity can also be enhanced with the use of a proper seeding strategy.^[^ ^31^ ^]^ Seeding is traditionally applied in industry to achieve appropriate crystal properties, such as high purity, desired morphology, polymorph, and uniform CSD.^[^ ^32^ ^]^ The factors that need to be considered for the successful design of a seeded crystallization process are the seed properties and amount of seed loading, seed addition technique, and crystallization control strategy.^[^ ^33^ ^]^ The use of a desired component as seed in solution also prevents uptake of impurity and enhances the product purity.

Lewis et al. (2022) reported the antisolvent crystallization of Nd sulfate at various organic to aqueous (O/A) ratios and impact of seeding on process yield and particle size distribution.^[^ ^34^ ^]^

In hydrometallurgical processing, iron removal without losses of valuable elements is often a challenge. For magnet recycling, oxidative roasting can be applied prior to leaching, whereby Fe is oxidized into Fe_2_O_3_, thereby facilitating selective leaching resulting in leach liquors with very low Fe to Nd ratios (often below 1 mol%). Antisolvent crystallization of REE from sulfuric acid provides an opportunity to obtain a pure REE solid concentrate from a leach solution with very high iron concentration due to the relatively high solubility of iron compared to REEs in the mixed solvent system; by this approach oxidative roasting can be avoided. Antisolvent crystallization of Nd_2_(SO_4_)_3_·8H_2_O and Pr_2_(SO_4_)_3_·8H_2_O in presence of Fe(II) and Fe(III) has been investigated in unseeded experiments with all antisolvent added directly in one pot.^[^ ^35^ ^]^ In this study the solubilities of FeSO_4_·H_2_O, Nd_2_(SO_4_)_3_·8H_2_O and Pr_2_(SO_4_)_3_·8H_2_O were investigated in 2.2 mol kg^−1^ H_2_SO_4_ aqueous and ethanol solutions in the O/A (mol/mol) range 0.1–0.7. Furthermore, the oxidation of Fe(II) to Fe(III) was investigated in 2.2 mol kg^−1^ H_2_SO_4_ aqueous ethanol solution with an O/A ratio of 0.5 (wt/wt). The results show that the oxidation of Fe(II) to Fe(II) is slow and that Nd and Pr tend to coprecipitate into mixed phases. Furthermore, the results indicate that crystals of Nd_2_(SO_4_)_3_·8H_2_O tend to be smaller and more agglomerated in presence of Fe(III) than in the presence of Fe(II) under the studied conditions. In this study, antisolvent crystallization of Nd and Dy sulphate hydrates was investigated in the presence of Fe(II) and Fe(III) as impurities to investigate the impact on the REE product quality in terms of purity and morphology. Both unseeded and seeded experiments were conducted. The generation and consumption of supersaturation was controlled by varying the addition rate, antisolvent concentration, and seed loading. Experiments were performed applying both magnetic and overhead stirring. Furthermore, the extent of impurity incorporation in pure and mixed phases of Nd and Dy, respectively, was investigated by laser ablation inductively coupled plasma mass spectrometry (LA‐ICP‐MS). The work gives insights on how to design an efficient process for recovery of pure REE concentrates from impure leach liquors, for example, in the recycling of magnet waste.

## 2 Experimental Section

Reagent grade Nd(III) oxide (Nd_2_O_3_) and Dy(III) oxide (Dy_2_O_3_) purchased from Treibacher Industries AG, 99.9%, iron (II) sulfate heptahydrate (Supelco.) and iron (III) sulfate hydrate (VWR) and Millipore water (having resistivity of 18.2 mΩ cm at 25 °C and a TOC < 5 ppb) were used to prepare solutions of the respective metals. Reagent grade ethanol (VWR) was used as an antisolvent.

The initial concentration of Nd(III), Dy(III), Fe(II), Fe(III), and sulfuric acid and the experimental conditions, including evaluated variables, are presented in **Table** **1** and **Table** **2**. The solubility of Nd and Dy sulfate hydrates in the aqueous phase was 25 and 20 g/L, respectively.^[^ ^36^, ^37^ ^]^ The solution compositions were chosen based on expected metal concentrations after leaching of NdFeB permanent magnet waste.^[^ ^38^ ^]^ These leach liquors will contain Nd(III) and Dy(III) together with relatively high amounts of iron. Depending on upstream treatment and chosen leaching conditions, the iron could be either in divalent or trivalent state. The oxidation of divalent iron to trivalent in aqueous ethanol solution at 25 °C was slow, obeying an exponential decay function with a half‐life of ≈600 h at an O/A ratio of 0.2 (mol mol^−1^) = 0.5 (wt/wt).^[^ ^35^ ^]^ The solubility of FeSO_4_·H_2_O in 2.2 mol kg^−1^ H_2_SO_4_ aqueous ethanol solutions had been determined by precipitation, that is, by approaching saturation from supersaturated conditions.^[^ ^35^ ^]^ By interpolation of the data given, the solubility in terms of total Fe(II) concentration at an O/A ratio of 0.5 (wt/wt) was 380 mmol kg^−1^ H_2_O (21.2 g kg^−1^). This was lower than the initial concentration of Fe(II) selected for the present study. However, the previous study indicates a long induction time for precipitation of FeSO_4_·H_2_O and slow kinetics. At an initial total iron(II) concentration of 30 g kg^−1^ no precipitation could be induced at an O/A ratio of 0.5, even after 14 days and addition of seeds.^[^ ^35^ ^]^ Furthermore, our previous work indicates that Fe(III) will not precipitate as a separate phase, nor lead to phase separation, under the chosen conditions.^[^ ^23^, ^35^ ^]^

**Table 1:** Initial concentration of Nd(III), Dy(III), Fe(II), and Fe(III) in the aqueous 2.2. mol/kg H_2_SO_4_ solution. The last column refers to seeding conditions connected to the respective initial solution compositions investigated.

Element	Conc. [g kg^−1^]	Conc. [mmol L^−1^]	Seedinga)
Nd(III)	8.0	54	None, Nd, Nd/Dy
Dy(III)	0.9	5.4	None, Nd, Nd/Dy
Fe(II) or Fe(III)	25	440	None, Nd, Nd/Dy
Fe(II) or Fe (III)	13	230	Nd, Nd/Dy
Nd(III)	7.0	49	Nd/Dy
Dy(III)	0.8	5.0	Nd/Dy
Fe(II) or Fe(III)	20	360	Nd/Dy

a) Seeding: None, Pure Nd_2_(SO_4_)_3_·8H_2_O (Nd), Mixed (Nd/Dy)_2_(SO_4_)_3_·8H_2_O (Nd/Dy).

**Table 2:** Experimental conditions.

Parameter	
Conc. H_2_SO_4_	2.2 mol kg^−1^ [2.0 mol L^−1^]
Final mass of antisolvent	41 g (52 mL)
Final O/A ratio	0.5, 0.2 (wt/wt)
Stirring speed (magnetic)	400 rpm
Stirring speed (overhead)	300 rpm
Antisolvent addition rate	0.1, 0.25, 1.0 [mL min^−1^]
Ageing timea)	24 h
Temperature	25 °C

a) Time after complete addition of antisolvent.

Experiments were carried out in 250 mL Erlenmeyer flasks with PTFE‐coated stirrer bars and agitated using submersible magnetic stirrers (2mag MIX drive 20 stirrer units with MIX control 15 controller units) immersed in a thermostatic water bath maintained at a temperature of 25 °C (Lauda LCB 4743 Model E 20G). The temperature in the bath was measured by a separate thermometer. Experiments were also performed in a jacketed crystallizer with a capacity of 150 mL using overhead stirring and maintained at a temperature of 25 °C using a thermostatic chiller (Julabo, Model MV‐4). All containers were sealed to avoid evaporation of solution. A syringe pump was used for controlled addition of antisolvent to the solution (Kd Scientific, Model number 200, Accuracy ± 0.35%). In each experiment 100 g of initial aqueous solution was used. Ethanol was used as an antisolvent. Ethanol was miscible with the aqueous solutions used in this study. It was also cheap and easily available, and the solubility of REEs was significantly reduced in the mixed water ethanol solvent system.^[^ ^22^ ^]^ Ethanol was added to the metal sulphate solutions at different addition rates until an O/A ratio of 0.5 (wt/wt) was reached. The time required to reach O/A 0.5 was 385, 163, and 52 min for an addition rate of 0.1, 0.25, 1 mL min^−1^, respectively, of 100% antisolvent. At an O/A ratio of 0.5 the solubility of Nd is low (1 g kg^−1^ H_2_O).^[^ ^35^ ^]^ After complete addition of antisolvent, the solution mixture was kept stirred for 24 h to allow the system to reach equilibrium.

For seeded trials, three types of seeds were prepared consisting of either pure Nd sulphate octahydrate (Nd_2_(SO_4_)_3_·8H_2_O) or a mixed Nd(III) and Dy(III) sulphate hydrate phase (solid solution). Two batches of mixed seeds were prepared. The composition of Dy in the mixed seeds was 10% of the Nd composition (mol/mol), which was confirmed using inductively coupled plasma optical emission spectroscopy (ICP‐OES) and energy dispersive X‐ray spectroscopy (EDX) analysis. The seeds were prepared in a similar manner to the antisolvent experiments using magnetic stirring, except that the experiment was terminated 10 min after complete addition of antisolvent (O/A ratio of 0.5). The temperature was 25 °C, the antisolvent dosing rate was 1 mL min^−1^, and the concentration of ethanol was 0.5 (wt/wt).

Seeded experiments were designed to avoid secondary nucleation and achieve conditions where supersaturation was consumed only by growth of the seed crystals. This could be achieved by balancing the rate of addition of antisolvent (concentration and dosing rate) and the consumption of supersaturation (seed loading).

The seed loading varied from 1 to 10%. During the initial 15 min, the antisolvent was added at 1 mL min^−1^ since the initial concentration of Nd was below the saturation concentration. When the system reached saturation, seed crystals were added and the antisolvent addition rate was reduced to 0.1 or 0.25 mL min^−1^.

Slurry samples (≈1 mL) were collected at different time intervals to determine the metal concentrations in the solution. The solution was immediately separated from the solid phase using a syringe filter (0.2 μm, PTFE), weighed, and diluted for subsequent analysis by ICP‐OES. After 24 h, the experiment was stopped, and the crystals were filtered from the mother liquor using a vacuum pump. The crystals were washed to remove the impurities. The washing solution (50 mL) had the same solvent composition as the mother liquor (2 mol L^−1^) sulfuric acid and ethanol at an O/A ratio of 0.5). The crystals were then dried at room temperature in the fume hood. The majority of experiments were performed in triplicate, and for these, average values with standard deviation were reported.

The recovery efficiency is calculated using Equation (1).

(1) $$\text{\% Recovery}\left(j\right)=\frac{C_{i}\left(j\right)m_{i}-C_{s}\left(j\right)m_{s}}{C_{i}\left(j\right)m_{i}}$$

where *C*_*i*_ and mi are the initial concentration (g kg^−1^) and mass (kg) of the solution respectively and *C*_*s*_ and ms is the final concentration (g kg^−1^) and mass of solution after 24 h.

For crystal size analysis, 100–200 μL of slurry sample was taken and placed on a glass slide and covered with a slip. Images were taken (at 10X or 5X magnification) using a camera connected to the microscope. The images were processed using ImageJ to determine the size distribution of crystals. For each sample 15–20 images were taken, and more than 300 crystals were analyzed to measure the area of crystals. The crystal diameter (mean size), determined from the measured areas, assume crystals of spherical shape. The mean crystal size was determined using Equation (2).

(2) $$D\left[43\right]=\frac{\sum n_{i}D_{i}^{4}}{\sum n_{i}D_{i}^{3}}$$

where *i* is the size of bin, *n*_*i*_ is the number of particles and *D*_*i*_ is the average size of particles.

Metal concentrations were determined using ICP‐OES (Thermo Scientific iCAP 7000 Plus) coupled to an autosampler (Cetac ASX‐520). Liquid samples were diluted using 1% v/v HNO_3_. Solid samples (0.5 g) were dissolved in water (50 mL), and the solution was diluted with 1% v/v HNO_3_ solution before ICP‐OES analyzes (0.5–50 mg L^−1^ range). An RF power of 1150 W was used. Triplicates were performed for each measurement, and results were reported as average values. The powder samples were also analysed with powder X‐ray diffraction (XRD) for phase identification and scanning electron microscopy with energy dispersive X‐ray spectroscopy (SEM‐EDX) to investigate the morphology, size distribution, and surface elemental composition of the crystals. Crystals had been analysed for Nd, Dy, and Fe by LA‐ICP‐MS at the GeoRessources Laboratory (Nancy, France). Laser ablation was performed with an ESI New Wave Research UC 193 nm excimer laser at a frequency of 10 Hz and a fluence of 6 J cm^−2^. A straight ablation with spot sizes of 60 μm was used. The ablated material was analyzed with an Agilent 7900 ICP‐MS and carried by helium gas (0.5 L min^−1^), which was mixed with argon (0.9 L min^−1^) via a cyclone mixer (volume of 9.5 cm^3^) before entering the ICP torch. External calibration was carried out using the NIST 610 and 612 glass standards. To calculate absolute concentrations, Nd was chosen as internal standard. Data reduction was performed using Iolite software (version 4)^[^ ^39^ ^]^ following the standard methods of Longerich et al. (1996).^[^ ^40^ ^]^

## 3 Results and Discussion

The XRD analysis shows the formation of Nd(III) sulphate octahydrate crystals (Nd_2_(SO_4_)_3_·8H_2_O) under all conditions applied (PDF‐card number 01‐089‐5176). The powder XRD patterns are reported in supporting information (S1).

### 3.1 Unseeded Experiments

The ethanol addition rate was 1 mL min^−1^, leading to a dosing time of 52 min for 100% and 116 min for 60% ethanol, respectively. The initial point of nucleation is difficult to observe with the naked eye. However, a clear spark of secondary nucleation was observed by the naked eye in the experiments with magnetic stirring and dosing 100% ethanol. This point of secondary nucleation occurred at a later point in time for the Fe(III) system (after 50 min) compared to the pure and Fe(II) systems (after 28 and 30 min, respectively).

**Figure 1:**
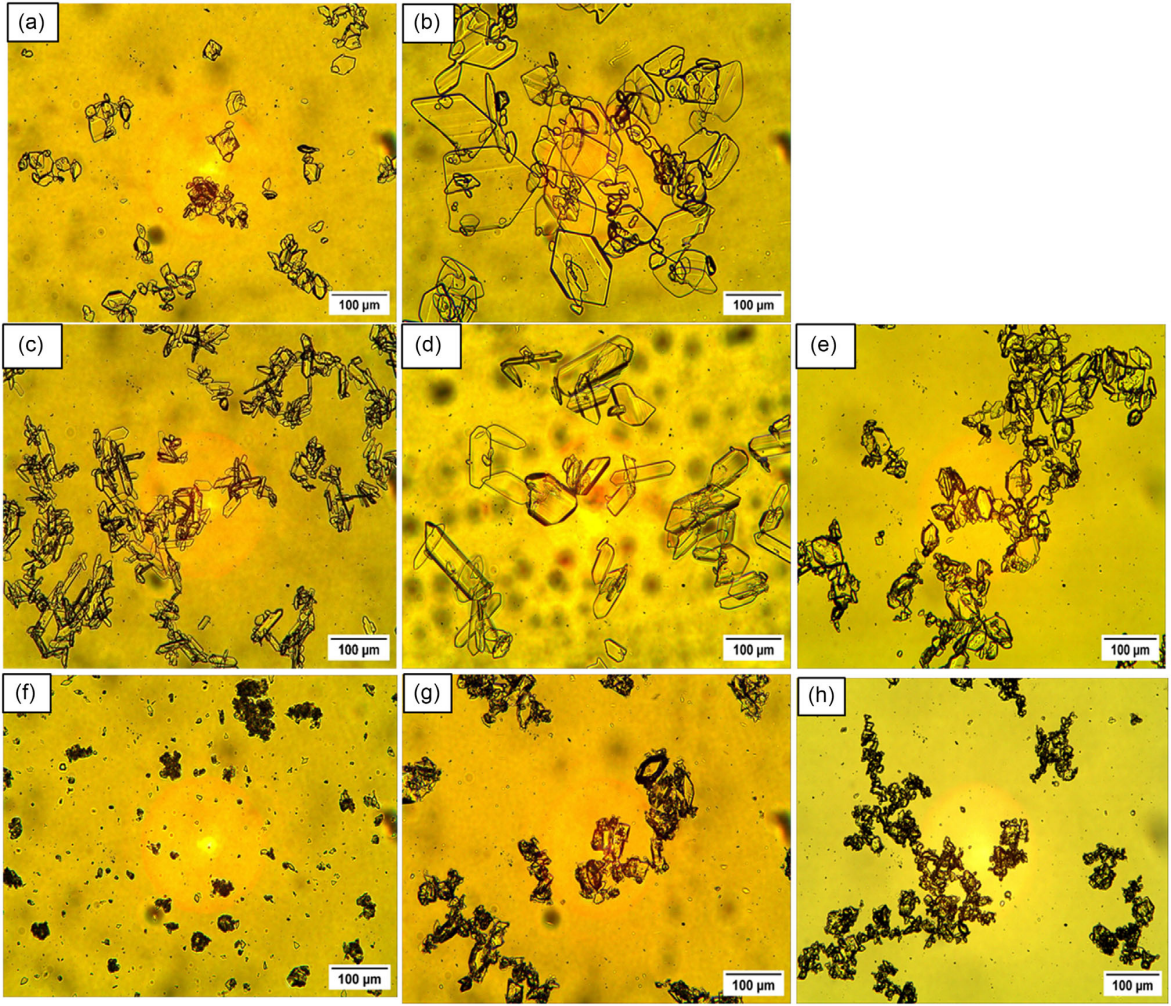
Micrograph of Nd_2_(SO_4_)_3_·8H_2_O crystals recovered from unseeded experiments with an antisolvent dosing rate of 1 mL min^−1^ for: solutions with no iron present applying a) magnetic stirring, b) overhead stirring after dosing 100% ethanol, solutions with 25 g kg^−1^ Fe(II) applying, c) magnetic stirring, d) overhead stirring for 100% ethanol addition, and e) magnetic stirring after dosing 60% ethanol and solutions with 25 g kg^−1^ Fe(III) applying, f) magnetic stirring, g) overhead stirring, and h) magnetic stirring after dosing 60% ethanol. The micrographs were taken at 10X magnification.

**Figure 2:**
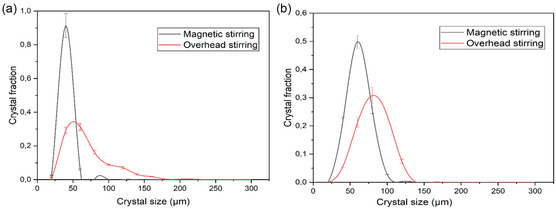
CSD plots of Nd_2_(SO_4_)_3_·8H_2_O crystals recovered from a) solutions with no iron present and from b) solutions with 25 g kg^−1^ Fe(II), applying magnetic stirring (black) or overhead stirring (red) respectively. In all cases 100% ethanol was added at 1 mL min^−1^.

Micrographs of the Nd_2_(SO_4_)_3_·8H_2_O crystals obtained at the end of the experiments are shown in **Figure** **1**, and CSD plots are shown in **Figure** **2**. The recovered crystals show hexagonal morphology, with smaller crystals resulting from the magnetically stirred experiment and larger crystals from overhead stirring. The difference between overhead and magnetic stirring carries significant implications for mixing efficiency due to different agitation and attrition conditions. The Reynolds number is 2730 and 2420 for the magnetic stirrer and overhead stirrer cases respectively, implying that mixing occurred in the turbulent mixing regimes. The tip speed, indicative of maximum shear, was determined as 0.73 m/s for the magnetic stirrer and 0.60 m/s for the overhead impeller, respectively. The power numbers were estimated taking into account the calculated suspension density and viscosity and solids fraction of 0.8 wt% (considering 100% recovery of Nd) using the Nagata correlation.^[^ ^41^ ^]^ The calculated power numbers for the magnetic stirrer and overhead impeller are 3.6 and 7.4, respectively, and the corresponding power inputs are 0.05 and 0.06 W, see **Table** **3**. Details of the correlations and parameters used are provided in the supporting information (S1). The power number and power input of the overhead‐stirred setup are larger than that of the magnetically stirred setup. Generally, a higher power number correlates with better mixing efficiency since more power is being supplied to the fluid by the impeller. However, different vessel and impeller design also impact the relationship between power number and mixing efficiency. Furthermore, magnetic stirrers will promote attrition and breakage more than overhead‐stirred vessels as a result of the high local shear between the stirrer bar and the vessel bottom surface,^[^ ^42^ ^]^ resulting in a relatively larger generation of smaller crystals.

**Table 3:** Mixing parameters for overhead and magnetic stirring.

Stirring mode	Diameter of Impeller [m]	Stirring speed [rpm]	Tip speed [m s^−1^]	Reynolds number	Power number	Power input [W]
Magnetic stirring	0.035	400	0.73	2 730	3.6	0.05
Overhead stirring	0.038	300	0.60	2 420	7.4	0.06

After addition of 100% ethanol the CSD plots show an average crystal size of 34 and 116 μm for magnetic and overhead stirring respectively in the pure system and 64 and 141 μm for magnetic stirring and overhead stirring respectively in the presence of Fe(II), see Figure 2. The crystal size considered is the length that is, the longest dimension. However, finer particles were neglected when measuring the crystal size distribution for all trials. The recovered crystals show a change in morphology to more elongated crystals in the presence of Fe(II) compared to the crystals obtained in the pure Nd system, for both magnetic and overhead stirring, see Figure 1. The Fe(II) could possibly affect the growth rate of the Nd_2_(SO_4_)_3_·8H_2_O crystal faces differently leading to elongated crystals. The crystals obtained while dosing diluted antisolvent are slightly larger and less elongated than those obtained dosing concentrated antisolvent. In the experiment adding diluted antisolvent, the supersaturation is lower during the duration of the experiment due to the addition of water, thus the crystals are expected to grow at a slower rate in these experiments. The crystals obtained in the presence of Fe(III) are smaller and more agglomerated, compared to the crystals obtained in the pure system and in the presence of Fe(II), see Figure 1. It was not possible to determine the crystal size by image analyzes because of the presence of agglomerates. The crystals obtained under overhead stirring and magnetic stirring by adding 60% ethanol are slightly larger than crystals recovered under magnetic stirring by using pure ethanol as antisolvent. This could be explained by nucleation of a fewer number of crystals at a lower supersaturation level, compared to the experiments when adding pure ethanol.

The crystals grown from solutions without iron and from solutions containing Fe(II) shows a high number of smaller sized crystals and a lower number of larger crystals (>100μm) resulting from the secondary nucleation observed by the naked eye in the beginning of the magnetically stirred experiments and by attrition. In contrast, the reduced secondary nucleation in overhead‐stirred experiments led to a wider crystal size distribution, see Figure 1 and 2.

**Figure 3:**
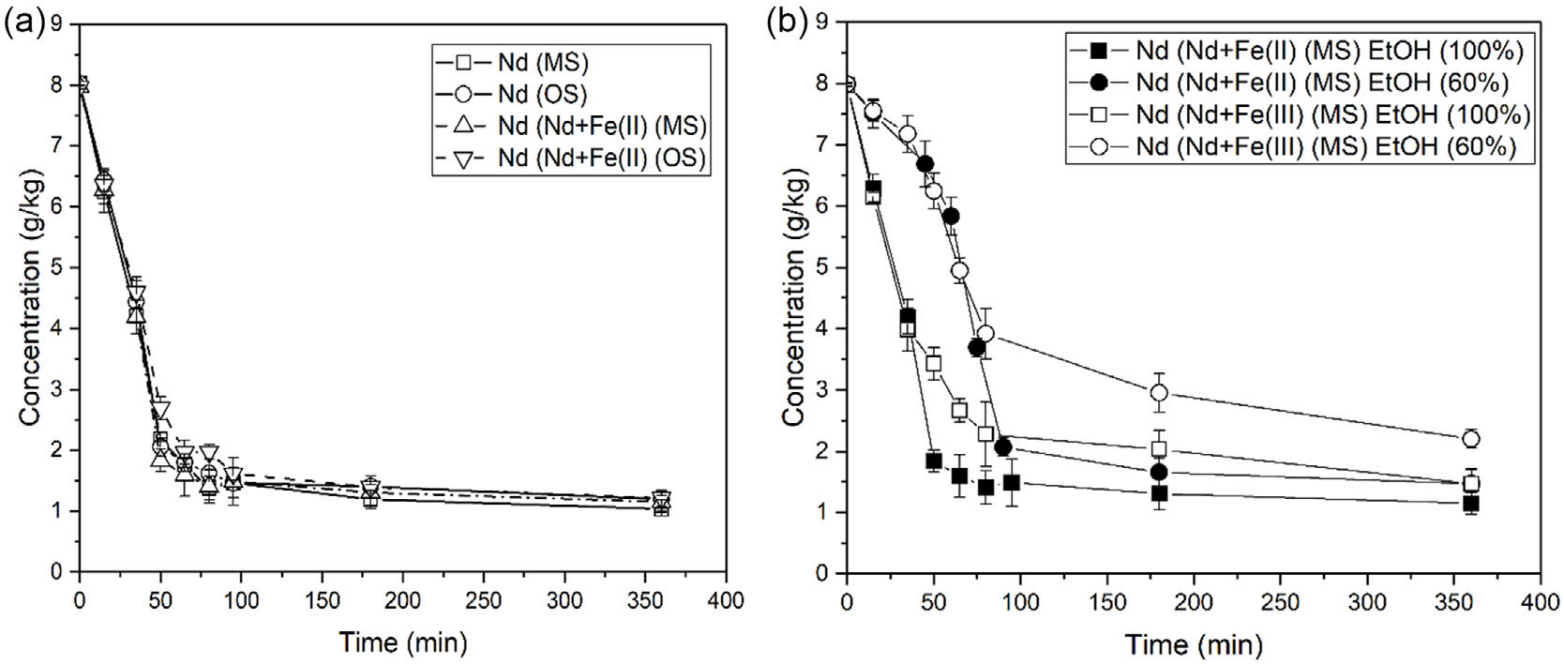
Desupersaturation profiles for Nd_2_(SO_4_)_3_·8H_2_O crystallized in unseeded experiments in the absence of iron and the presence of 25 g kg^−1^ of Fe(II) or Fe(III) with a) addition of 100% EtOH applying either magnetic or overhead stirring and b) addition of 100% or 60% EtOH applying magnetic stirring. The antisolvent addition rate was 1 mL min^−1^. The error bars represent deviation from the average value for three repeated experiments. Conc. is expressed as mass per kg initial solution.

The decrease in concentration of Nd(III) as a function of time is similar when no iron is present and in the presence of Fe(II), both for magnet and overhead stirring, see **Figure** **3**a. The slight difference in crystal size does not seem to have a distinguishable impact on the rate of consumption of the supersaturation due to crystal growth. The desupersaturation profiles show a slower decrease in Nd concentration versus time when dilute antisolvent is added compared to pure antisolvent, see Figure 3b. This is due to the additional water added to the solution, leading to a comparably lower rate of antisolvent addition and also a dilution of the solution by water which increases the solubility of Nd and thus decreases the supersaturation driving force for crystal growth. The decrease in Nd(III) concentration as a function of time is slower in the presence of Fe(III) than in the presence of Fe(II) even though the crystals are smaller in the presence of Fe(III), see Figure 3b. These results indicate that Fe(III) hinders the growth rate of Nd_2_(SO_4_)_3_·8H_2_O more than Fe(II) and promotes agglomeration of the crystals.

The Fe(II) and Fe(III) concentrations as a function of time are shown in the supporting information (S1). The Fe(II) concentration decreases when pure ethanol is added (3.22 g kg^−1^), when diluted ethanol is added, the decrease is lower (0.36 g kg^−1^), indicating that more iron is precipitated when concentrated ethanol is added. The concentration of Fe(II) detected after the end of the experiments when pure ethanol is added (21.8 g kg^−1^) is close to the solubility of FeSO_4_·H_2_O in 2.2 mol kg^−1^ H_2_SO_4_ aqueous ethanol solutions with O/A = 0.5 (wt/wt) (21.2 g kg^−1^) determined by precipitation.^[^ ^35^ ^]^ A lower decrease in Fe(III) concentration (2.2 g kg^−1^) is detected compared to the experiments with Fe(II). When diluted ethanol is added no decrease in Fe(III) could be detected.

### 3.2 Seeded Experiments

#### 3.2.1 Seed Characterization

**Figure 4:**
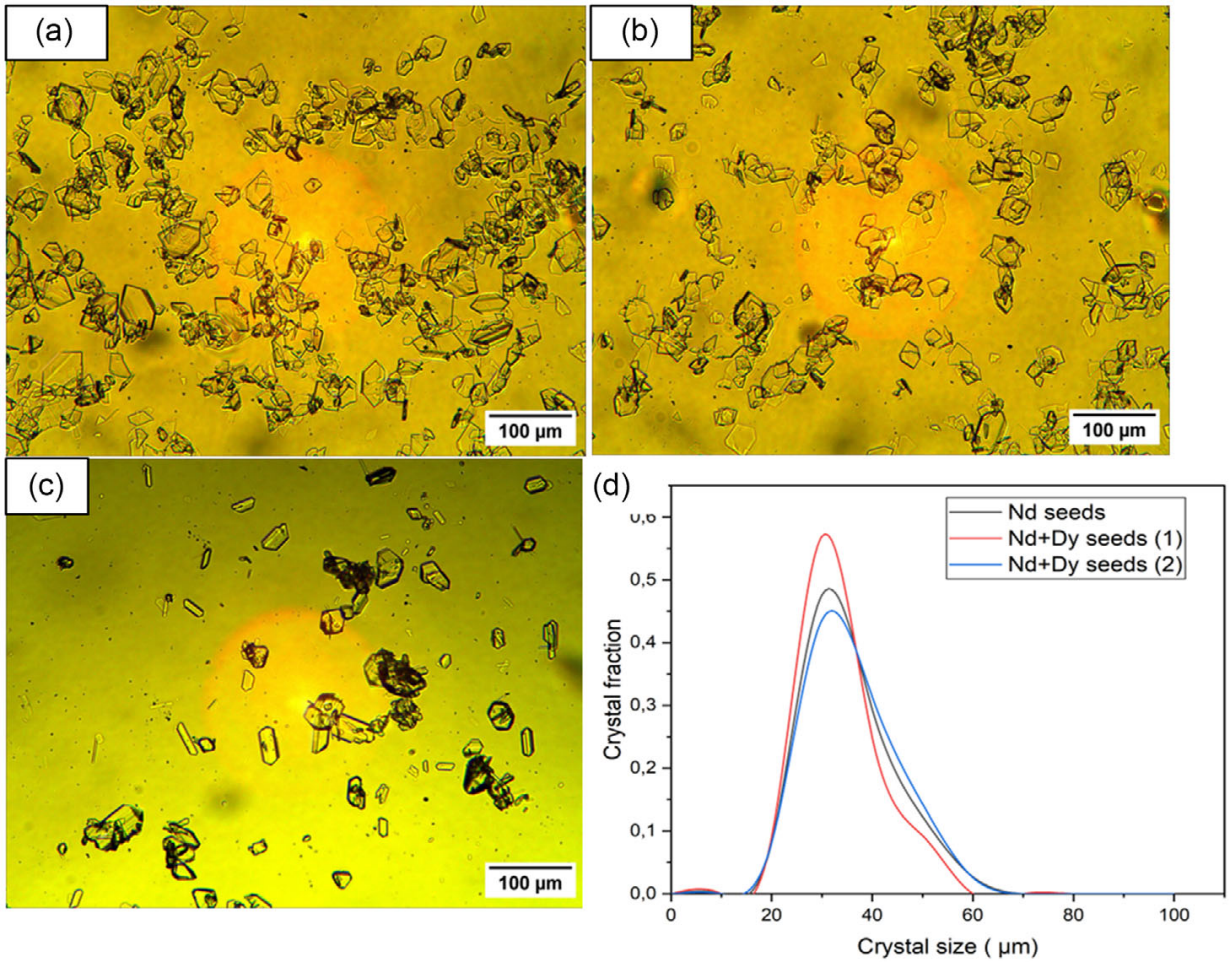
a) Micrographs of the seeds of Nd_2_(SO_4_)_3_·8H_2_O and of (Nd/Dy)_2_(SO_4_)_3_·8H_2_O, b) batch 1 and c) batch 2, and d) CSD plot of the seeds of Nd_2_(SO_4_)_3_·8H_2_O (a). The micrographs were taken at 10X magnification.

Micrographs of the synthesized pure Nd and mixed Nd/Dy seed crystals and their CSD profiles are shown in **Figure** **4**. Two batches of Nd/Dy seeds were prepared with the same composition, see Figure 4b,c. The seeds contained 10% by mass of Dy, which was confirmed by SEM‐EDX analysis (supporting information, Table 1). The presence of Dy in the seed crystals does not change the crystal structure as the same powder XRD patterns were obtained in both cases, that is, Nd_2_(SO_4_)_3_·8H_2_O (see Figure S7, Supporting Information). The mean size of seed crystal obtained are 37, 35 μm and 34 μm for Nd, Nd+Dy (1) and Nd+Dy seeds (2), respectively, displaying similar characteristics, see Figure 4d.

#### 3.2.2 Seeded Antisolvent Crystallization

To find the conditions where growth could be promoted over secondary nucleation, experiments were conducted with different seed loading and antisolvent addition rates applying magnetic stirring. Seed loadings of 1%, 2.5%, 5%, and 10% were chosen using the pure seeds of Nd_2_(SO_4_)_3_·8H_2_O. Ethanol (100%) was added at a rate of either 0.1 mL min^−1^ or 0.25 mL min^−1^. The solutions contained 25 g kg of Fe(II). The concentration of neodymium and iron as a function of time, micrographs of the crystals, and CSD plots are presented in the supporting information (S1). Secondary nucleation occurs when there are not enough crystals present in the system to consume the supersaturation generated by adding antisolvent. The experiments with a seed loading of 10% favored crystal growth with reduced secondary nucleation. Micrographs reported for different seed loading shows larger size crystals for 10% seed loading, see Figure S10, Supporting Information. Furthermore, the crystals are comparably larger for the slower addition rate at the same seed loading, indicating promotion of growth over nucleation under these conditions. The optimum seed loading of 10% and an addition rate of 0.1 mL min^−1^ of antisolvent was chosen for the rest of the seeded experiments.

**Figure 5:**
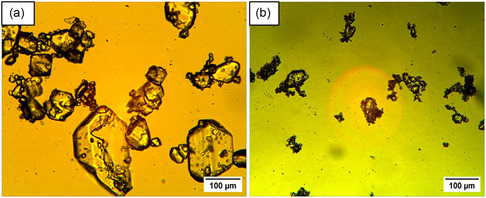
Micrographs of Nd_2_(SO_4_)_3_·8H_2_O obtained from solutions containing a) 25 g kg^−1^ Fe(II) and b) 25 g kg^−1^ Fe(III). Seeded crystallization was performed applying magnetic stirring with a seed loading of 10% Nd_2_(SO_4_)_3_·8H_2_O and an addition rate of 0.1 mL min^−1^ of concentrated ethanol. The micrographs were taken at 10X magnification.

Micrographs of Nd_2_(SO_4_)_3_·8H_2_O obtained from solutions containing 25 g kg^−1^ Fe(II) and 25 g kg^−1^ Fe(III) respectively in seeded experiments under magnet stirring are shown in **Figure** **5**. The crystals obtained in the presence of Fe(III) are smaller and more agglomerated, compared to the crystals obtained in the presence of Fe(II). The crystals observed in presence of Fe(III) resembles the crystals obtained in the unseeded experiments, presented in Figure 4f.

**Figure 6:**
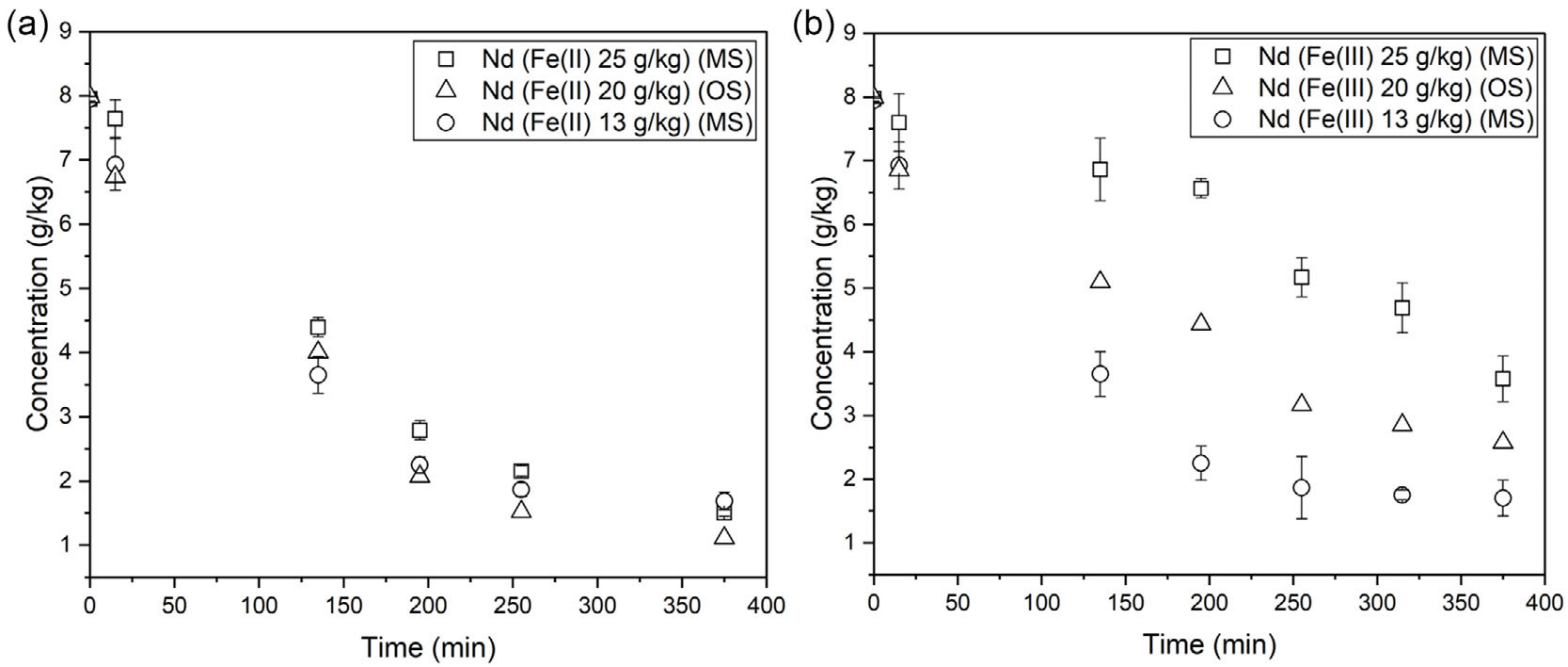
Desupersaturation curves of Nd for growth of Nd_2_(SO_4_)_3_·8H_2_O in the presence of different amounts of a) Fe(II) and b) Fe(III). Seeded crystallization was performed applying magnetic and overhead stirring with a seed loading of 10% Nd_2_(SO_4_)_3_·8H_2_O and an addition rate of 0.1 mL min^−1^ of pure ethanol. Conc. is expressed as mass per kg initial solution to correct the value from any dilution effects due to ethanol addition.

The desupersaturation curves for neodymium in experiments with 13 and 25 g kg of Fe(II) and Fe(III) applying magnetic stirring and 20 g kg^−1^ of Fe(II) and Fe(III) applying overhead stirring are shown in **Figure** **6**. There is no large impact on the desupersaturation curves of Nd(III) as the Fe(II) concentration varies between 13 and 25 g kg^−1^, see Figure 6a. In the presence of Fe(III), the concentration of neodymium decreases slowly by time as the concentration of Fe(III) increases, see Figure 6b, even though the crystals are smaller than in the presence of Fe(II). The results indicate that Fe(III) hinders the growth rate of Nd_2_(SO_4_)_3_·8H_2_O more than Fe(II), in accordance with the findings from the unseeded experiments. There are several mechanisms through which impurities can affect the growth rate of Nd_2_(SO_4_)_3_·8H_2_O. They can adsorb onto the crystal surface, blocking active growth sites and preventing solute ions from attaching. They may also be incorporated into the crystal lattice, causing defects and strain that disrupt the orderly growth process. In some cases, impurities compete with solute ions for adsorption, reducing the efficiency of growth. Additionally, impurities can form complexes with solute ions, lowering the free ion concentration in solution and thus decreasing the supersaturation needed for growth. Lastly, impurities may modify electrostatic interactions between ions and the crystal surface, further inhibiting growth by altering how ions attach to the crystal.^[^ ^43^, ^44^, ^45^ ^]^ In this case, it could be due to adsorption of Fe(III) onto Nd_2_(SO_4_)_3_·8H_2_O blocking active growth sites, causing reduction in crystal growth. The decrease in concentration of Fe(II) and Fe(III) as a function of time are reported in supporting information (S1).

**Figure 7:**
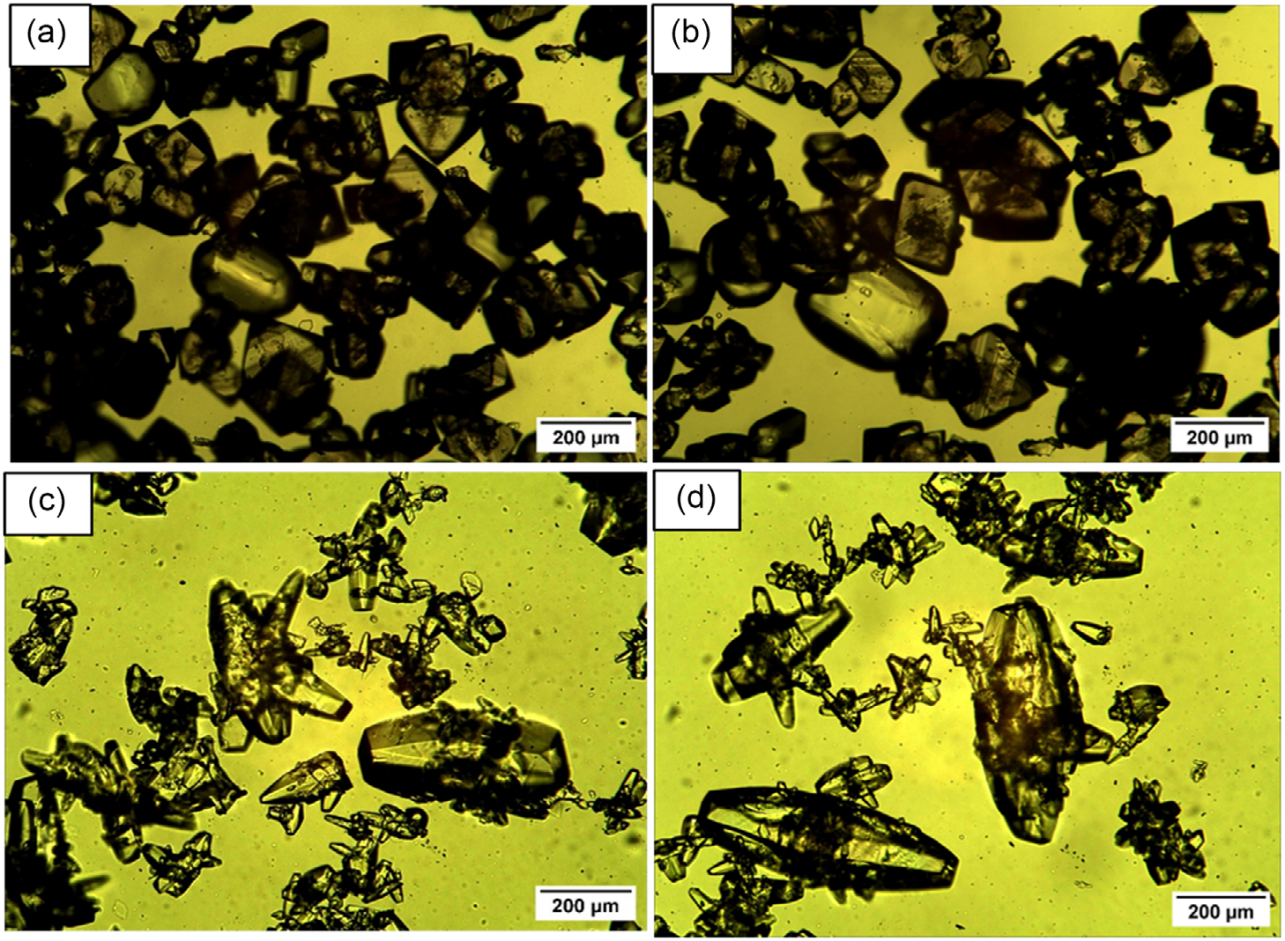
Micrographs of the crystals recovered from solutions with a) 25 g kg^−1^ of Fe(II) and 10% Nd seed loading, b) 10% Nd+Dy seed loading, and from solution with 25 g kg^−1^ of Fe(III) and c) 10% Nd seed loading, d) 10% Nd+Dy seed loading. The antisolvent addition rate was 0.1 mL min^−1^ and overhead stirring was applied. The micrographs were taken at 5X magnification.

Experiments were performed to crystallize Nd_2_(SO_4_)_3_·8H_2_O with 10% seed loading of either Nd_2_(SO_4_)_3_·8H_2_O or (Nd/Dy)_2_(SO_4_)_3_·8H_2_O applying overhead stirring. The ethanol addition rate was 0.1 mL min^−1^, and the initial iron concentration was 25 g kg^−1^ of either Fe(II) or Fe(III). The decrease in concentration of neodymium and iron as a function of time is similar for seeding with pure and mixed crystals, respectively, see Figure S8, Supporting Information. The mean crystal size of Nd_2_(SO_4_)_3_·8H_2_O grown in the presence of Fe(II) was 170 μm after seeding with pure Nd seeds and 185 μm after seeding with mixed Nd and Dy seeds, see CSD plots (Figure S11, Supporting Information). This indicates that both Nd_2_(SO_4_)_3_·8H_2_O and (Nd/Dy)_2_(SO_4_)_3_·8H_2_O act equally good as seeds for growth of Nd_2_(SO_4_)_3_·8H_2_O under these conditions. The crystals are larger when overhead stirring is used compared to experiments under the same conditions applying magnetic stirring, see Figure 5 and **Figure** **7**. No secondary nucleation was observed in the overhead‐stirred experiments in the presence of Fe(II), see Figure 7a,b. The micrographs of the crystal samples obtained in the presence of Fe(III) show larger crystals surrounded by smaller crystals both when seeding with pure and mixed seeds, see Figure 7c,d.

Less agglomeration is observed for overhead stirring compared to magnetic stirring. Smaller particles have a higher tendency to agglomerate than larger particles.^[^ ^46^ ^]^ The crystals obtained in the overhead‐stirred experiments are larger than the crystals from the magnetically stirred experiments which can explain the decreased degree of agglomeration.

**Figure 8:**
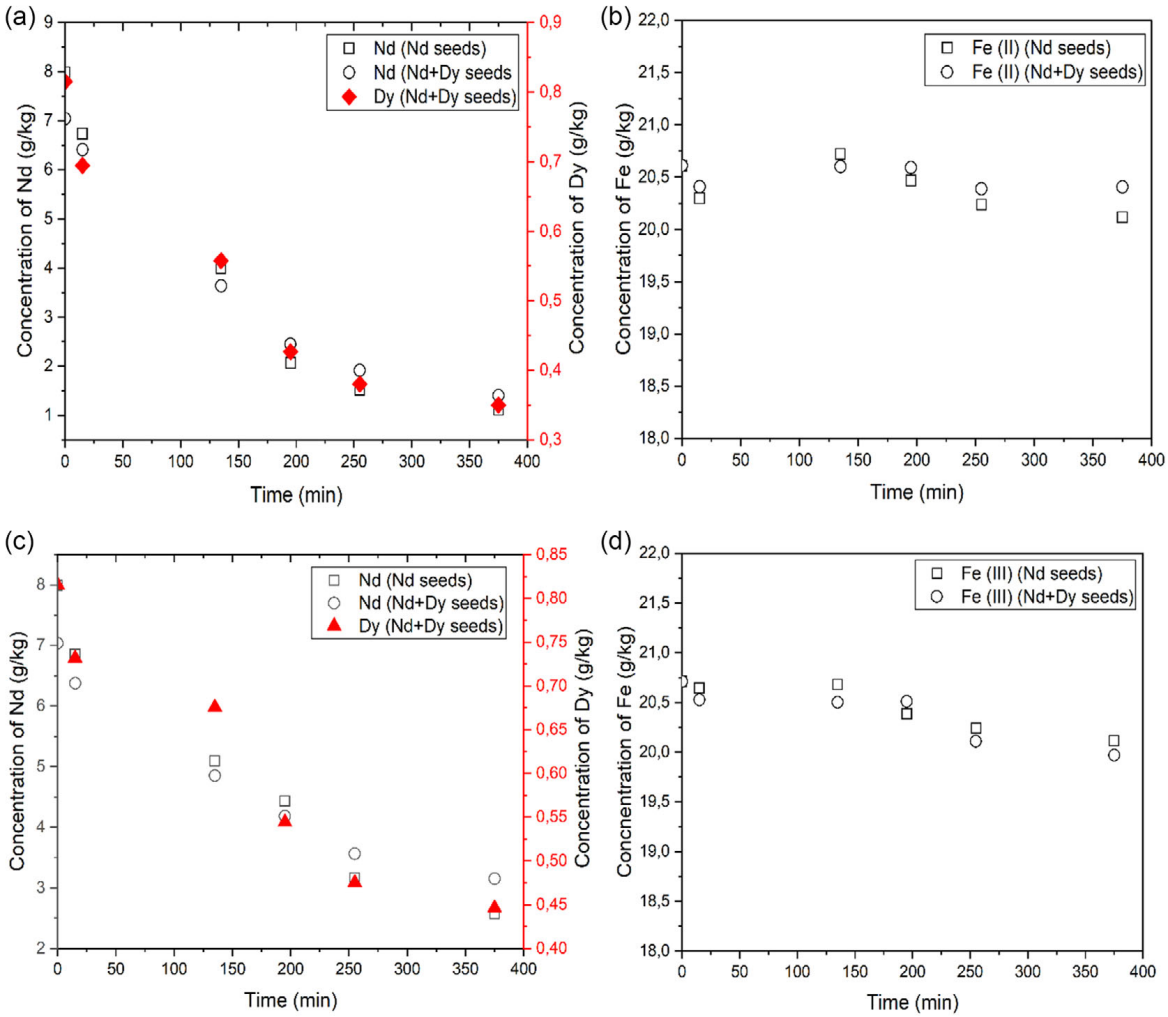
Desupersaturation profile for Nd and Dy with time for 10% Nd and Nd+Dy seed loading respectively with an initial concentration of 20 g kg^−1^ of Fe(II) showing the decrease in concentration of a) Nd and Dy and b) Fe(II) as a function of time and with an initial concentration of 20 g kg^−1^ of Fe(III) showing the decrease in concentration of c) Nd and Dy, and d) Fe(III) as a function of time. The antisolvent addition rate was 0.1 mL min^−1^ applying overhead stirring. Conc. is expressed as mass per kg initial solution.

The incorporation of Fe(II) and Fe(III) into Nd_2_(SO_4_)_3_·8H_2_O and (Nd/Dy)_2_(SO_4_)_3_·8H_2_O after seeding with the respective phase was studied under conditions of lower iron concentration (20 g kg^−1^) to reduce the probability of crystallizing iron as a separate phase.^[^ ^35^ ^]^ Overhead stirring was applied in these experiments. The combined initial concentration of Nd and Dy (54 mol kg^−1^) in solution was equal to the total concentration of Nd in the experiments where pure Nd_2_(SO_4_)_3_·8H_2_O was crystallized to make them better comparable in terms of supersaturation. The concentration of neodymium, dysprosium and iron as a function of time are presented in **Figure** **8**. The decrease in concentration of Nd is similar in the pure and mixed systems and the decrease in Dy concentration is proportional to the decrease in Nd concentration. The molar fraction of Dy in relation to Nd crystallized at each point in time evens out around 7.5% after 135 min. For the experiment with Fe(III) the relative fraction of Dy to Nd in solution evens out around 9% after 375 min. The molar ratio of Dy/Nd in the solid phases reflect the molar concentrations present in the liquid phases and the seed composition.

**Figure 9:**
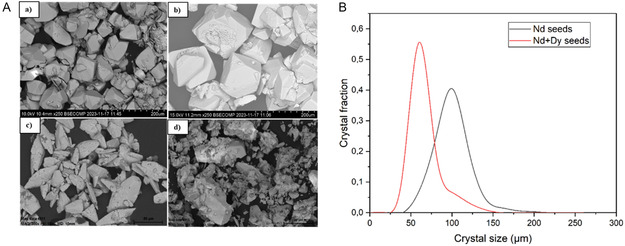
A) SEM images of a) (Nd/Dy)_2_(SO_4_)_3_·8H_2_O and b) Nd_2_(SO_4_)_3_·8H_2_O obtained in the presence of 20 g kg^−1^ of Fe(II), and c) (Nd/Dy)_2_(SO_4_)_3_·8H_2_O and d) Nd_2_(SO_4_)_3_·8H_2_O obtained in the presence of 20 g kg^−1^ of Fe(III). The antisolvent addition rate was 0.1 mL min^−1^ and overhead stirring was applied. B) Crystal size distributions in crystallization of Nd_2_(SO_4_)_3_·8H_2_O (black) and (Nd/Dy)_2_(SO_4_)_3_·8H_2_O (red) respectively in the presence of Fe(II).

Images of the respective crystals are presented in **Figure** **9**A. The crystals are similar to Nd_2_(SO_4_)_3_·8H_2_O crystallized in the presence of 25 g kg^−1^ Fe(II) and Fe(III), respectively presented in Figure 7. The mixed (Nd/Dy)_2_(SO_4_)_3_·8H_2_O crystals (Figure 9a) are distinctly smaller than the pure Nd_2_(SO_4_)_3_·8H_2_O crystals (Figure 9b) obtained from solutions containing 20 g kg^−1^ of Fe(II), see Figure 9B. The sample of Nd_2_(SO_4_)_3_·8H_2_O crystals obtained in the presence of 20 g kg^−1^ of Fe(III) (Figure 9d) shows larger crystals surrounded by smaller crystals, while the (Nd/Dy)_2_(SO_4_)_3_·8H_2_O crystal sample (Figure 9c) contains fewer smaller crystals. This indicates a more excessive secondary nucleation in the pure neodymium experiment compared to the mixed neodymium and dysprosium experiment in the presence of Fe(III).

### 3.3 Washing of Crystals

The purity of crystals in unseeded and seeded experiments in the presence of either Fe(II) or Fe(III) was investigated before and after washing, and the results are presented in **Table** **4**. The wash solution had the same solvent composition as the mother liquor. The results show that washing greatly improves the purity of the solid phase in the case of Fe(II), while washing only has a small impact on the purity of the solid phase in the case of Fe(III); this indicates an increased tendency for Fe(II) to precipitate as separate iron crystals compared to Fe(III).

**Table 4:** Purity of Nd_2_(SO_4_)_3_·8H_2_O crystals before and after washing. The crystals were obtained in experiments after addition of concentrated (99.95%) ethanol under magnetic stirring. The purity was analyzed by ICP‐OES after dissolution.

Seeding	Sample	Fe(II) [g kg^−1^]	Purity [%]
Nd_2_(SO_4_)_3_·8H_2_O	Before wash	114 ± 9	69 ± 0.6
	After wash	2 ± 1	99.5 ± 0.2
Unseeded	After wash	20 ± 3	94 ± 0.76

Seeding	Sample	Fe(III) [g kg^−1^]	Purity [%]
Nd_2_(SO_4_)_3_·8H_2_O	Before wash	3 ± 2	99.2 ± 0.3
	After wash	3 ± 1	99.2 ± 0.4
Unseeded	After wash	6 ± 2	98.3 ± 0.5

**Figure 10:**
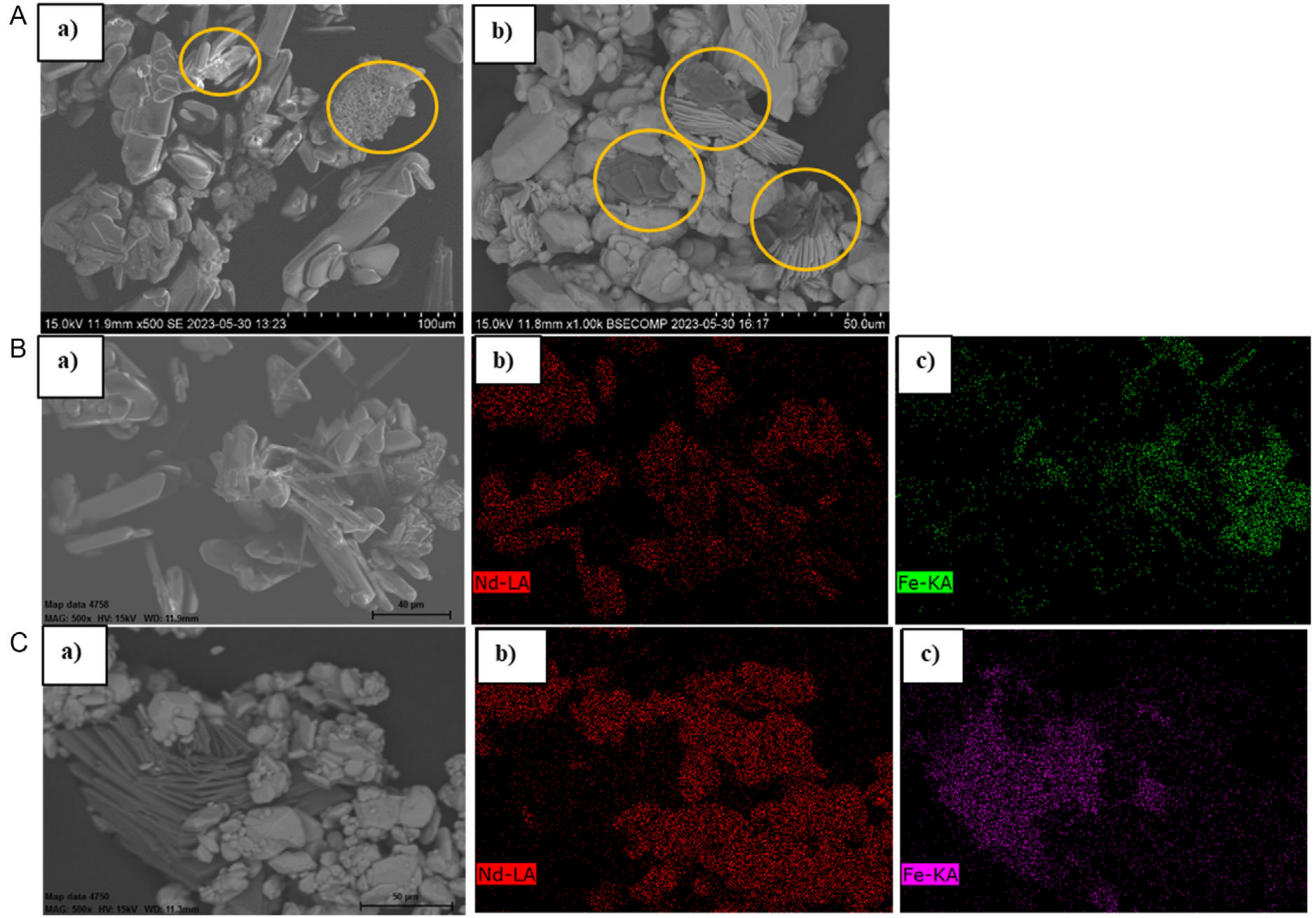
SEM images for washed crystals recovered from unseeded experiments with an addition rate of 1 mL min^−1^ concentrated antisolvent and applying magnetic stirring A) a) Nd_2_(SO_4_)_3_·8H_2_O, Fe(II) and b) Nd_2_(SO_4_)_3_·8H_2_O, Fe(III), B) EDX mapping of Nd_2_(SO_4_)_3_·8H_2_O, Fe(II), and C) EDX mapping of Nd_2_(SO_4_)_3_·8H_2_O, Fe(III).

SEM‐EDX analysis of washed crystals obtained in unseeded experiments shows the presence of Fe(II) in separate granular solids (**Figure** **10**A(a)) and Fe(III) as plate‐shaped solids (Figure 10A(b)), see Figure 10A. The surface elemental composition of Fe(II) and Fe(III) detected with EDX mapping is presented in supporting information S1.

**Figure 11:**
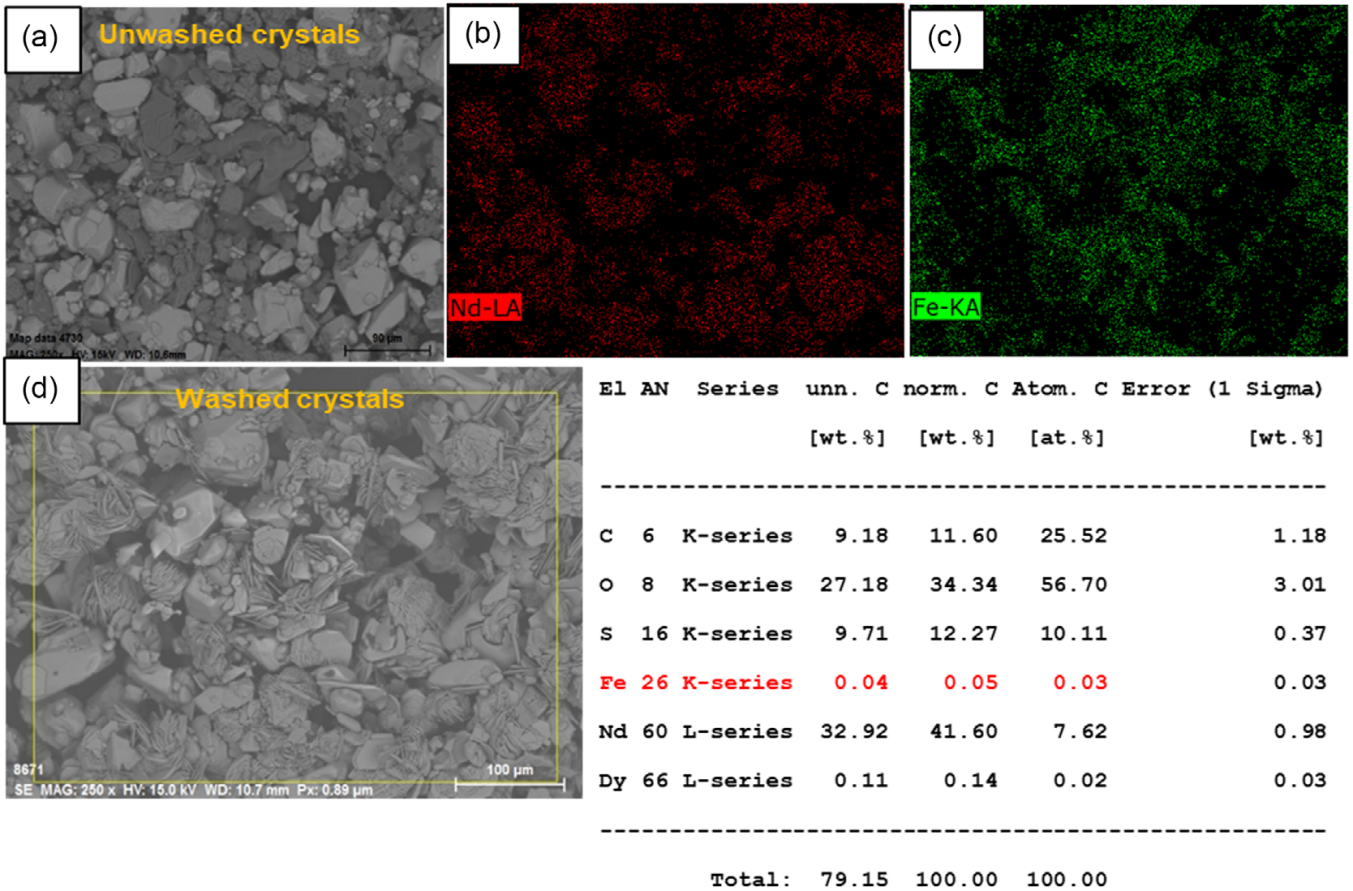
a,b,c) EDX mapping of unwashed Nd_2_(SO_4_)_3_·8H_2_O crystals recovered from 10% Nd seed loading and elemental composition of washed crystals, and d) initial Fe(II) concentration of 25 g kg^−1^ and an antisolvent addition rate of 0.1 mL min^−1^ and applying magnetic stirring.

**Figure 12:**
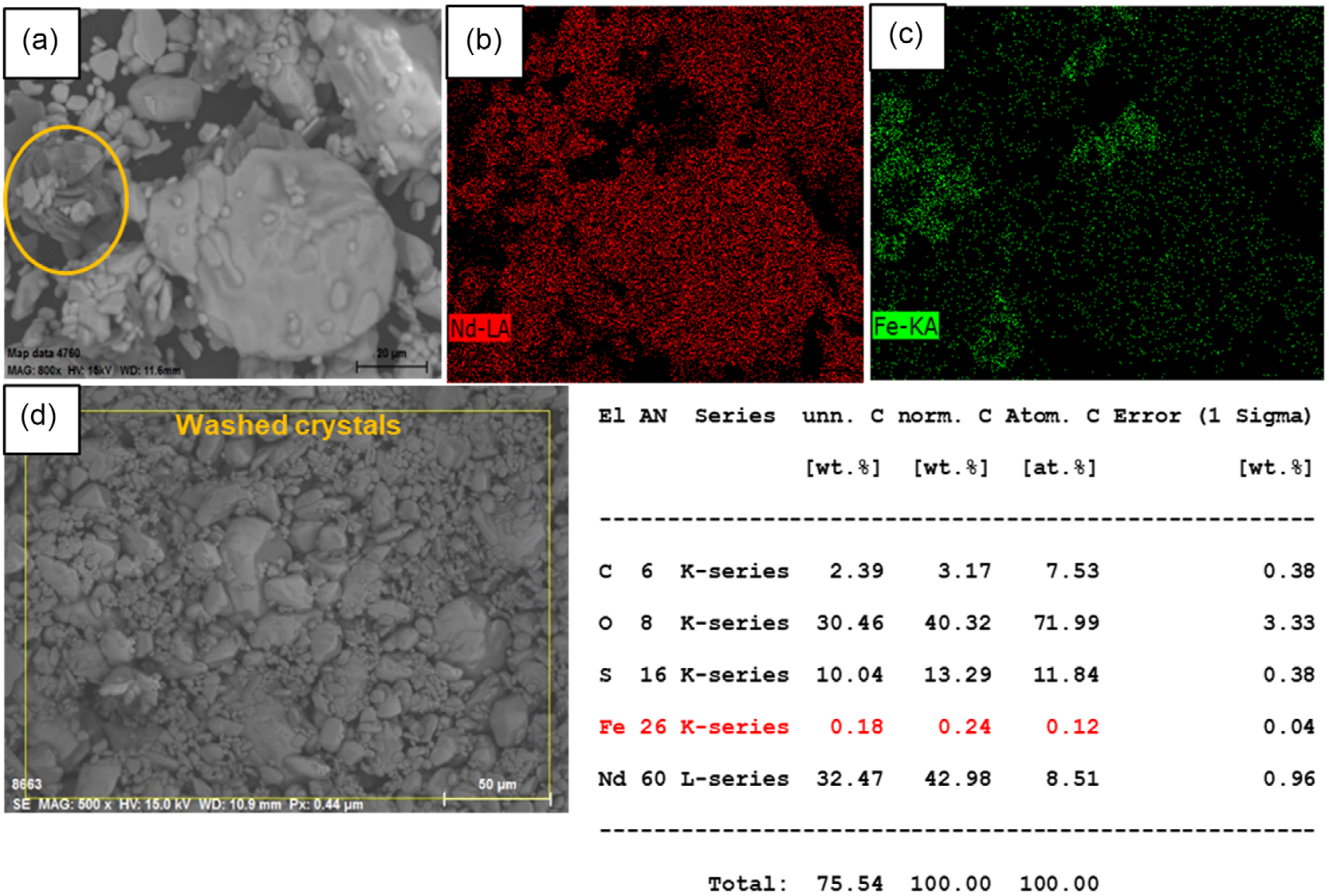
a,b,c) EDX mapping of unwashed Nd_2_(SO_4_)_3_·8H_2_O crystals recovered from 10% Nd seed loading and elemental composition of washed crystals, and d) initial Fe(III) concentration of 25 g kg^−1^ and an antisolvent addition rate of 0.1 mL min^−1^ and applying magnetic stirring.

**Figure 13:**
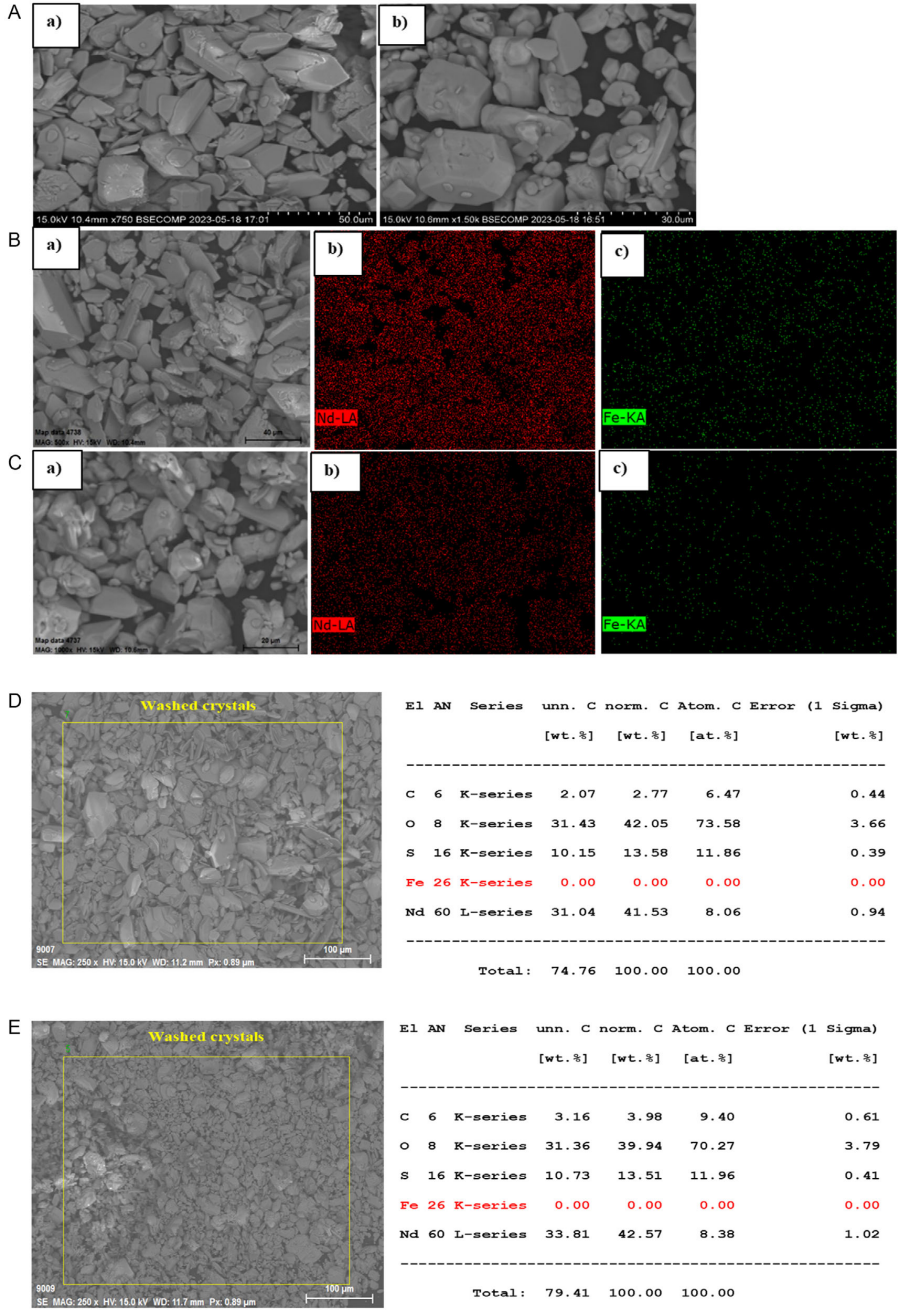
SEM images of washed Nd_2_(SO_4_)_3_·8H_2_O crystals recovered from unseeded trials with an addition rate of 1 mL min^−1^ of diluted antisolvent (60%) and applying magnetic stirring A) a) Fe(II) and b) Fe(III). B and D) EDX mapping of crystals containing Nd and Fe(II) and C and E) EDX mapping of crystals containing Nd and Fe(III).

SEM images of unwashed crystals obtained in seeded experiments in the presence of Fe(II) and Fe(III) and magnetic stirring show the presence of two crystal morphologies, see **Figure** **11**,**Figure** **12**, respectively. The larger crystals mainly contain neodymium, whereas iron is found to be concentrated in smaller clusters of solid captured in the EDX mapping. In the washed crystals, iron is present at lower concentration. The EDX mapping of the washed crystals (Figure 11d and 12d) results in a similar iron content as detected by redissolving the crystals (0.1–0.5 wt%, see Table 4 and **Table** **5**). In the sample crystallized in the presence of Fe(II) a low content of Dy is detected (0.1 wt%), see Figure 11d. SEM images of washed crystals after adding diluted antisolvent (60%) show no separate Fe crystals and a low and even distribution of Fe in the sample, see **Figure** **13**.

**Table 5:** The purity of washed Nd_2_(SO_4_)_3_·8H_2_O crystals obtained in unseeded and seeded experiments with addition of concentrated (100%) ethanol under magnetic and overhead stirring.

REE	Initial Fe(II) conc. [g kg^−1^]	Set‐up	Antisolvent	Addition rate [mL min^−1^]	Seed loading	Seed type	Fe(II) in solid [g kg^−1^]b)	Purity [%]a)
Nd	25	Magnetic	100% ethanol	1	–	–	20 ± 3	94.5 ± 0.4
Nd	25	Overhead	100% ethanol	1	–	–	21 ± 3	94.2 ± 0.3
Nd	25	Magnetic	60% ethanol	1	–	–	n.d.	100%
Nd	25	Magnetic	100% ethanol	0.1	1%	Nd	9 ± 2	97.6 ± 0.3
Nd	25	Magnetic	100% ethanol	0.1	2.5%	Nd	7 ± 2	97.8 ± 0.4
Nd	25	Magnetic	100% ethanol	0.1	5%	Nd	4 ± 3	98.9 ± 0.6
Nd	25	Magnetic	100% ethanol	0.1	10%	Nd	2 ± 1	99.4 ± 0.2
Nd	25	Magnetic	100% ethanol	0.25	5%	Nd	13 ± 3	96.7 ± 0.5
Nd	25	Magnetic	100% ethanol	0.25	10%	Nd	17 ± 4	95.7 ± 0.6
Nd	25	Magnetic	100% ethanol	0.1	10%	Nd+Dy	3 ± 1	99.1 ± 0.2
Nd	25	Overhead	100% ethanol	0.1	10%	Nd	2 ± 1	99.5 ± 0.2
Nd	25	Overhead	100% ethanol	0.1	10%	Nd+Dy	3 ± 1	99.2 ± 0.2
Nd	13	Magnetic	100% ethanol	0.1	10%	Nd	n.d.	100
Nd	13	Magnetic	100% ethanol	0.1	10%	Nd+Dy	n.d.	100
Nd	20	Overhead	100% ethanol	0.1	10%	Nd	0.2	99.9
Nd, Dyc)	20	Overhead	100% ethanol	0.1	10%	Nd+Dy	0.1	99.9

REE	Initial Fe(III) conc. [g kg^−1^]	Set‐up	Antisolvent	Addition rate [mL min^−1^]	Seed loading	Seed type	Fe (III) in solid [g kg^−1^]b)	Purity [%]a)
Nd	25	Magnetic	100% ethanol	1	**–**	**–**	6 ± 2	98.3 ± 0.5
Nd	25	Overhead	100% ethanol	1	**–**	**–**	5 ± 4	98.2 ± 0.4
Nd	25	Magnetic	60% ethanol	1	**–**	**–**	n.d.	100%
Nd	25	Magnetic	100% ethanol	0.1	10%	Nd	3 ± 1	99.2 ± 0.2
Nd	25	Magnetic	100% ethanol	0.1	10%	Nd+Dy	3 ± 2	99.2 ± 0.4
Nd	25	Overhead	100% ethanol	0.1	10%	Nd	3 ± 2	99.2 ± 0.4
Nd	25	Overhead	100% ethanol	0.1	10%	Nd+Dy	4 ± 2	98.9 ± 0.4
Nd	13	Magnetic	100% ethanol	0.1	10%	Nd	n.d.	100
Nd	13	Magnetic	100% ethanol	0.1	10%	Nd+Dy	n.d.	100
Nd	20	Overhead	100% ethanol	0.1	10%	Nd	3	99.1
Nd, Dyd)	20	Overhead	100% ethanol	0.1	10%	Nd+Dy	2	99.2

a) Purity is reported as mass‐% Nd of the total metal mass in the crystals.

b) n.d. = not detected

c) Purity is represented in terms of total REE mass‐% (Dy content is 8.2%).

d) Purity is represented in terms of total REE mass‐% (Dy content is 9.1 %).

### 3.4 Solid Phase Purity

The purity of the washed Nd_2_(SO_4_)_3_·8H_2_O crystals obtained in the unseeded experiments are reported in Table 5. The results show that adding dilute (60%) ethanol results in higher purity than when concentrated (100%) ethanol is added. The crystals show similar purity regardless of setup used (magnetic and overhead‐stirred setups). Furthermore, the results indicate that Fe(II) incorporates to a larger extent in the solid product compared to Fe(III). Since these data are obtained after a total dissolution of the respective sample, it is not possible to determine to which extent the iron is present as separate crystals and/or incorporated into the neodymium phase.

The purity of the Nd_2_(SO_4_)_3_·8H_2_O crystals obtained in the presence of Fe(II) and Fe(III) respectively, is higher for seeded experiments than for the unseeded experiments, see Table 5. For the experiments conducted at the addition rate of 0.1 mL min^−1^ the purity improves as the seed loading increases from 1% to 10%. A lower addition rate of ethanol resulted in higher purity. The highest purity is obtained for the highest seed loading (10%) and lowest addition rate (0.1 mL min^−1^). No difference in purity of Nd_2_(SO_4_)_3_·8H_2_O and (Nd/Dy)_2_(SO_4_)_3_·8H_2_O can be detected in samples where pure Nd seeds and mixed Nd and Dy seeds are used, respectively under the conditions investigated for magnetic and overhead‐stirred experiments. In the experiments with the lowest initial concentration of Fe(II) and Fe(III) of 13 g kg^−1^ no iron could be detected in the solid phase by ICP‐OES analysis after dissolving the crystals. The systematic change in purity with seed loading and antisolvent addition rate indicates that the Fe(II) is primarily introduced into the sample during the crystallization process rather than during the washing stage. Since these data are obtained after a total dissolution of the respective sample, it is not possible to determine to which extent the iron is present as separate crystals or incorporated into the neodymium phase, respectively.

The molar fraction of Dy in the mixed Nd/Dy crystals was 8% and 9% grown in the presence of Fe(II) and Fe(III), respectively. This correlates well with the measured relative decrease in concentration of Dy and Nd at the end of the experiments, where the molar ratio is 7.5% (Fe(II)) and 9.0% (Fe(III)) respectively, see Figure 8.

Crystals of Nd_2_(SO_4_)_3_·8H_2_O and (Nd/Dy)_2_(SO_4_)_3_·8H_2_O obtained in seeded experiments with an initial iron concentration of 20 g kg^−1^ with overhead stirring were analyzed by laser ablation inductively coupled plasma mass spectroscopy (LA‐ICP‐MS). The results are presented in **Table** **6**. The concentrations are reported as g metal per kg total mass of crystal. Iron was only detected in the crystals obtained after crystallizing Nd_2_(SO_4_)_3_·8H_2_O in the presence of Fe(III). In all other samples, the iron concentration was below the detection limit (<8 mg kg^−1^). This indicates that Fe(III) is incorporated into the main phase of Nd_2_(SO_4_)_3_·8H_2_O but not of (Nd/Dy)_2_(SO_4_)_3_·8H_2_O, while Fe(II) is not incorporated in either of the solid phases. The Fe(III) content obtained using LA‐ICP‐MS varies at different points. This significant difference of Fe(III) content in Nd_2_(SO_4_)_3_·8H_2_O could be due to uneven distribution of impurity throughout the crystals phase, unlike Dy which has almost similar content and is distributed uniformly in Fe(II) and Fe(III) trials (Table 6). Further investigation is needed for detailed understanding of Fe(III) incorporation in the Nd_2_(SO_4_)_3_·8H_2_O phase.

**Table 6:** LA‐ICP‐MS analyzes of washed crystals obtained in seeded experiments (10% seed loading) with an initial iron concentration of 20 g kg^−1^ after addition of concentrated (99.95%) ethanol at 0.1 mL min^−1^ with overhead stirring.

REE	Iron valency	Seed type	Dy [g kg^−1^]a)	Fe [g kg^−1^]a)
Nd	Fe(II)	Nd	n.d.	n.d.
Nd	Fe(II)	Nd	n.d	n.d.
Nd	Fe(II)	Nd	n.d	n.d
Nd, Dy	Fe(II)	Nd+Dy	12.1 ± 0.84	n.d.
Nd, Dy	Fe(II)	Nd+Dy	10.60 ± 0.5	n.d.
Nd, Dy	Fe(II)	Nd+Dy	9.60 ± 0.73	n.d.
Nd, Dy	Fe(II)	Nd+Dy	9.19 ± 0.48	n.d.
Nd	Fe(III)	Nd	n.d.	3.76 ± 0. 64
Nd	Fe(III)	Nd	n.d.	30.57 ± 4.49
Nd	Fe(III)	Nd	n.d.	48. 84 ± 5.47
Nd, Dy	Fe(III)	Nd + Dy	11.53 ± 1.46	n.d.
Nd, Dy	Fe(III)	Nd + Dy	12.79 ± 0.63	n.d.
Nd, Dy	Fe(III)	Nd + Dy	11.55 ± 0. 87	n.d.

a) n.d.: not detected (<8 mg kg^−1^ Fe).

### 3.5 Concluding Discussion

Iron is seen to have an impact on the morphology and growth rate of Nd_2_(SO_4_)_3_·8H_2_O and (Nd/Dy)_2_(SO_4_)_3_·8H_2_O crystals. The key observations are summarized in **Table** **7**. Under certain conditions, more elongated crystals of Nd_2_(SO_4_)_3_·8H_2_O are obtained in the presence of Fe(II). Furthermore, the results indicate that Fe(III) hinders the growth of Nd_2_(SO_4_)_3_·8H_2_O more than Fe(II) and promotes agglomeration of the crystals. Less agglomeration is observed for overhead stirring compared to magnetic stirring, which can be explained by the larger size of the particles in the overhead‐stirred experiments.

**Table 7:** Key observations on the impact of ferric and ferrous iron on the crystallization of Nd_2_(SO_4_)_3_·8H_2_O and (Nd/Dy)_2_(SO_4_)_3_·8H_2_O.

Condition	Fe(II)	Fe(III)
Nucleation	No detected impact on nucleation of the Nd_2_(SO_4_)_3_·8H_2_O crystals.	Prolonged time until nucleation is detected by the naked eye (50 min) compared to the pure Nd system (28 min).
Growth	No detected impact on the growth rate of the Nd_2_(SO_4_)_3_·8H_2_O crystals (Figure 3a and 6a).	Reduced growth rate of the Nd_2_(SO_4_)_3_·8H_2_O crystals, the rate decreases as the concentration of Fe(III) increases (Figure 3b and 6b).
Size and morphology	Formation of more elongated crystals of Nd_2_(SO_4_)_3_·8H_2_O for unseeded trials at a relatively high antisolvent addition rate of 1 mL min^−1^. No change in morphology in seeded experiments conducted at lower antisolvent addition rates of 0.25 and 0.1 mL min^−1^.	Reduced crystal size. Smaller crystals tend to agglomerate.
Impurity incorporation	Fe(II) precipitates as separate crystals. No Fe(II) could be detected in the Nd and Nd/Dy crystals.	Fe(III) is incorporated as separate crystals and is detected in the Nd crystals, unevenly distributed, but not in the Nd/Dy crystals.

The effective ionic radii of trivalent Nd (0.983 Å) and Dy (0.912 Å) for a coordination number of 6 are similar, and REE tend to form solid solutions, which is also seen in the present study.^[^ ^47^, ^48^, ^49^ ^]^ Furthermore, both Nd_2_(SO_4_)_3_·8H_2_O and (Nd/Dy)_2_(SO_4_)_3_·8H_2_O act equally good as seeds for growth of Nd_2_(SO_4_)_3_·8H_2_O.

The systematic change in purity with seed loading and antisolvent addition rate indicate that Fe(II) is primarily introduced into the sample during the crystallization process rather than during the washing stage. Fe(II) incorporates to a larger extent in the solid product compared to Fe(III) by precipitation as a separate phase. However, only Fe(III) is detected in the solid crystals of Nd_2_(SO_4_)_3_·8H_2_O. Fe(III) is seen to hinder the growth of Nd_2_(SO_4_)_3_·8H_2_O which indicate adsorption to the growing crystals. The stability of the surface complex determines the growth kinetics while the removal of the complex determines the capability of incorporation of the impurity; the balance between these processes determine the value of the effective segregation coefficient of the impurity at a specific level of supersaturation.^[^ ^50^ ^]^ In order to be incorporated the impurity must meet charge and size requirements. Under the applied conditions Fe(II) and Fe(III) likely exist as high‐spin, 6‐coordinate species due to the weak field strength of water, ethanol, and sulphate ligands, which favor high‐spin complexes. In 6‐coordination and high‐spin Fe(II) has an effective ionic radius of 0.780 Å and Fe(III) has an effective ionic radius of 0.645 Å.^[^ ^48^ ^]^ The ionic radius of Fe(II) is therefore more comparable to that of Nd(III) and Dy(III). However, the shared trivalent charge increases the likelihood that Fe(III) can substitute for Nd(III) or Dy(III) in the crystal lattice. Iron can also be incorporated as liquid inclusions and further work is recommended to determine how iron is present in the REE host crystals.

In the design of an efficient process for recovery of pure REE concentrates from impure leach liquors, it is important to avoid conditions of locally high supersaturation in the crystallization and washing stages since this can lead to precipitation of separate iron phases. This is especially critical if iron is present in a divalent state. However, only Fe(III) is seen to be incorporated into the solid phase of Nd_2_(SO_4_)_3_·8H_2_O. Interestingly, Fe(III) is only detected in the pure Nd_2_(SO_4_)_3_·8H_2_O phase and not in the mixed (Nd/Dy)_2_(SO_4_)_3_·8H_2_O phase. Furthermore, a more excessive secondary nucleation is observed in the crystallization of Nd_2_(SO_4_)_3_·8H_2_O compared to the mixed phase under equivalent conditions, indicating a more hindered growth of the pure phase. An extended investigation of this observation is recommended.

## 4 Conclusions

Seeding, regardless of whether using pure Nd_2_(SO_4_)_3_·8H_2_O or a mixed (Nd/Dy)_2_(SO_4_)_3_·8H_2_O seed (containing 10% Dy), effectively promotes Nd_2_(SO_4_)_3_·8H_2_O growth and can yield high‐purity products (>99%). However, iron impurities significantly influence the crystallization process. Specifically, Fe(III) markedly hinders crystal growth and exhibits a higher tendency for incorporation into the Nd_2_(SO_4_)_3_·8H_2_O phase (though not into the mixed (Nd/Dy)_2_(SO_4_)_3_·8H_2_O phase), as confirmed by LA‐ICP‐MS. In contrast, Fe(II) primarily affects crystal morphology, inducing elongation under certain conditions, and is not incorporated into either crystal phase. Practically, these results underscore the critical importance of minimizing Fe(III) contamination during rare earth element recovery from sulfate solutions to ensure high product purity and efficient crystallization. Furthermore, controlled antisolvent addition and adequate seed loading are crucial for limiting supersaturation and maximizing product purity. Future research should focus on finding the precise mechanisms of Fe(III) incorporation and developing optimized crystallization strategies, including antisolvent dosing and seed management, to mitigate impurity effects and enhance the efficiency and robustness of industrial‐scale rare earth separation processes.

## Conflict of Interest

The authors declare no conflict of interest.

## Supporting information

Supplementary Material

## Data Availability

The data that support the findings of this study are available from the corresponding author upon reasonable request.
