# Supplementary material for: Impact of Ferric and Ferrous Iron on the Crystallization of Rare Earth Sulphate Hydrates
Source: ChemSusChem. 2025 Jul 17;18(16):e202500285. doi: 10.1002/cssc.202500285 (PMC12330332; doi:10.1002/cssc.202500285)
Supplement: Supplementary file 1 — Supplementary Material [file CSSC-18-e202500285-s001.pdf]

Supporting information (S1)

Impact of Iron on the Crystallization of Rare Earth Sulphate Hydrates

3. Results and Discussion

3.1. Controlled addition experiments

3.1.1 Single component system: Nd

The XRD analysis shows the formation of Nd (III) sulphate octahydrate crystals ($Nd_2(SO_4)_3{\cdot}8H_2O$) in set A (magnetic stirring) and set B (overhead stirring) type of experiments under all conditions applied (PDF-card number 01-075-1729). The PXRD patterns are reported in Figure S1.

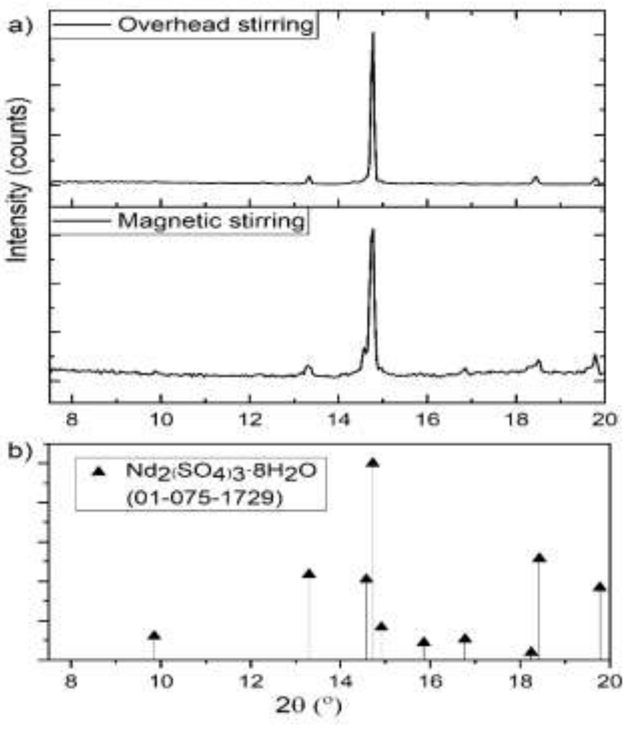

Figure S1. a) PXRD pattern for Nd (III) sulphate octahydrate crystals ($Nd_2(SO_4)_3{\cdot}8H_2O$) performed under Magnetic and Overhead stirring. Antisolvent addition rate was 1 mL/min. b) reference peaks for ($Nd_2(SO_4)_3{\cdot}8H_2O$) phases (PDF card numbers given in legends)

The desupersaturation curves of Nd (III) in the experiments using magnetic (set A) and overhead stirring (set B) respectively and a dosing rate of concentrated ethanol of 1 mL/ min is shown in Figure S2. The antisolvent was dosed for 52 min, time zero is when the addition of antisolvent starts. The Nd concentration decreases during the first 100 min of the

experiment and then slowly reaches equilibrium conditions by the end of the experiment. The recovery efficiency at an O/A ratio of 0.5 was 86 % and 84% in magnetic and overhead stirring experiments respectively.

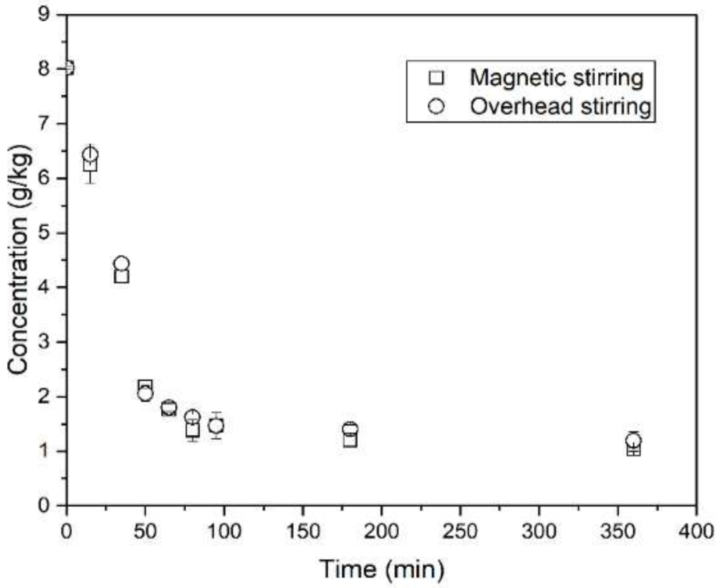

Figure S2. Nd concentration as a function of time in the experiments using magnetic (set A) and overhead (set B) stirring respectively. The error bars represent deviation from average value for three repeated experiments. Conc. is expressed as mass per kg initial solution.

3.1.2 Effect of mode of stirring:

The difference between overhead and magnetic stirring carries significant implications for mixing efficiency due to different agitation and attrition conditions. Nagata's correlations were used to calculate the power numbers were estimated taking into account the calculated suspension density and viscosity, and solids fraction of 8 wt% (considering 100% recovery of Nd) and a liquid depth of 45 mm in a jacketed vessel of diameter 60 mm for overhead stirring and, a liquid depth of 50 mm in an Erlenmeyer flask of diameter 50 mm for magnetic stirring.

$$N_p = \frac{A}{Re} + B\left(\frac{1000+1.2Re^{0.66}}{1000+3.2Re^{0.66}}\right)^{C} \times \left(\frac{h}{D_T}\right)^{\left(0.35+\frac{b}{D_T}\right)} \times sin\theta^{1.2} \qquad (1)$$

$$A = 14 + \frac{b}{D_T}(670\left(\frac{d}{D_T} - 0.6\right)^2 + 185 \qquad (2)$$

$$B = 10^{\left(1.3-4\left(\frac{b}{D_T}-0.6\right)^2 - 1.14\left(\frac{d}{D_T}\right)\right.} \qquad (3)$$

$$C = 1.1 + 4\left(\frac{b}{D_T}\right) - 2.5\left(\frac{d}{D_T} - 2.5\right)^2 - 7\left(\frac{b}{D_T}\right)^4 \quad (4)$$

Where $Re = \frac{Nd^2\rho}{\mu}$ ie the Reynolds number; h the height of liquid level, D_T is the diameter of the vessel, b is the width of the impeller blade, d is diameter of the impeller or stirrer bar, θ is the angle between agitator blade surface and horizontal surface, taken as 90° in this case, A,B, and C are parameters that are related to the system geometry.

3.1.3 Binary component system: Nd+Fe

3.1.3.1 Nd+Fe (II)

The neodymium concentration as a function of time is plotted in Figure S3a. Experiments were conducted using magnetic stirring (set A) with 100% and 60% ethanol respectively and using overhead stirring (set B) and 100% ethanol addition. The ethanol addition rate was 1 mL/min leading to a dosing time of 52 min for 100% ethanol and 116 min for 60% ethanol respectively. The desupersaturation profiles show a slower decrease in Nd concentration as a function of time in the experiment using dilute antisolvent compared to when 100% ethanol is added, see Figure S3a. The recovery efficiency was 88 and 86% for magnetic and overhead stirring respectively after addition of concentrated ethanol, showing a similar trend as in the experiment with no iron present. When adding diluted antisolvent the recovery efficiency was 80% due to the slightly higher amount of water added in this experiment. The iron (II) concentration as a function of time is shown in Figure S3b. The iron concentration in the solution decreases during the experiment. The measured decrease in iron concentration is larger in the experiments where concentrated ethanol was added (3.22 g/kg) compared to the experiment when diluted ethanol is dosed to the solution (0.36 g/kg), indicating that more iron is precipitated when concentrated ethanol is added compared to when diluted antisolvent is added.

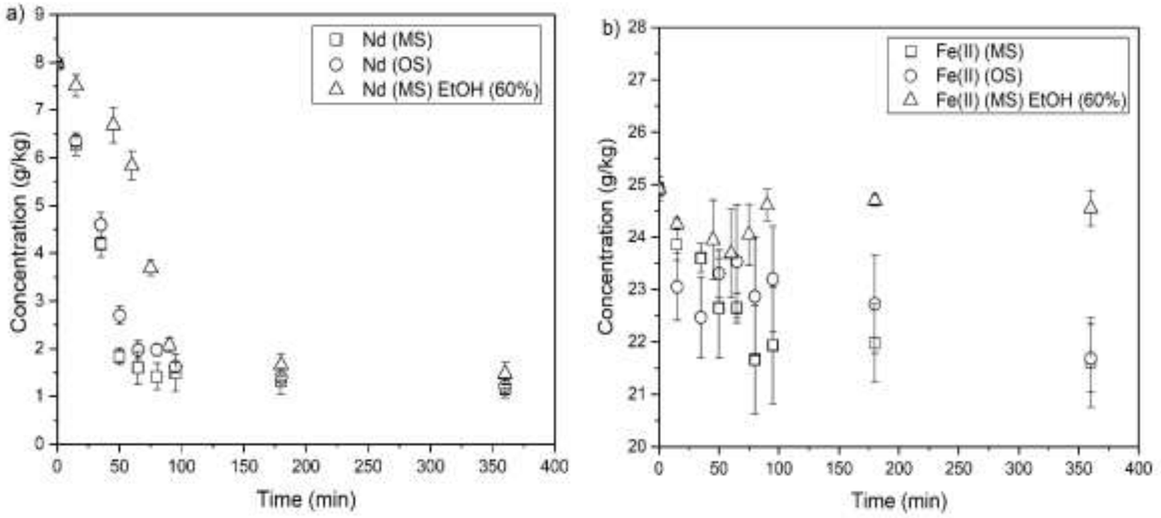

Figure S3 . Desupersaturation profiles of Fe II in adding concentrated EtOH under magnetic stirring (MS) and overhead stirring (OS) and 60% ethanol using magnetic stirring at 1 mL/min of antisolvent addition rates. Conc. is expressed as mass per kg initial solution.

The crystals were identified as $Nd_2(SO_4)_3{\cdot}8H_2O$ by powder XRD, see Figure S4.

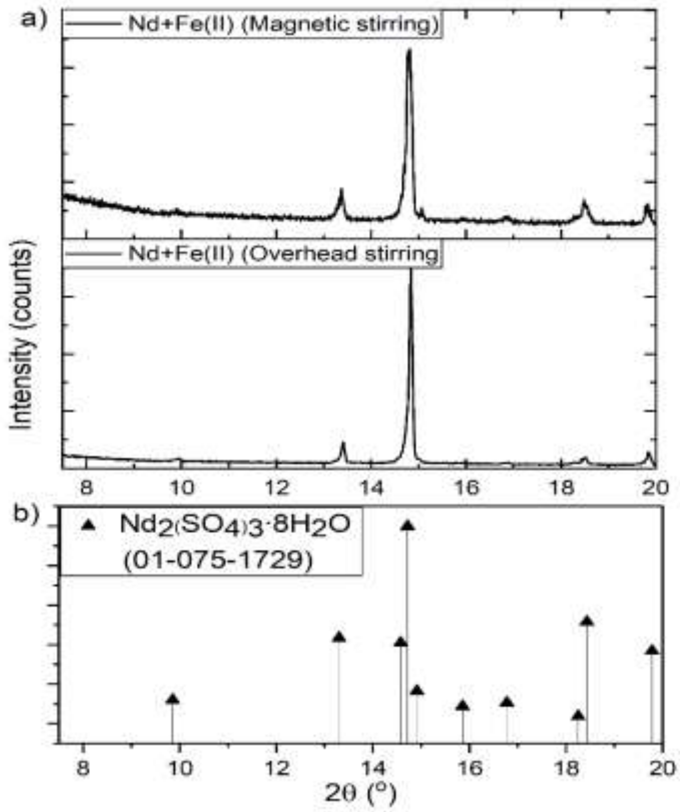

Figure S4. a) PXRD pattern for Nd+Fe (II) trial performed under Magnetic and Overhead stirring. Antisolvent addition rate was 1 mL/min. b) reference peaks for ($Nd_2(SO_4)_3{\cdot}8H_2O$) phases (PDF card numbers given in legends)

3.1.2.2 Nd+Fe (III)

The decrease in neodymium concentration as a function of time is shown in Figure S5a. Experiments were conducted using magnetic stirring (set A) with 100% and 60% ethanol respectively and using overhead stirring (set B) and 100% ethanol addition. The ethanol addition rate was 1 mL/min leading to a dosing time of 52 min for 100% and 116 min for 60% ethanol respectively. The desupersaturation profiles show a slower decrease in Nd concentration by time when dilute antisolvent is added compared to concentrated antisolvent. The neodymium recovery efficiency is 84% after 24h in the experiments adding 100% ethanol and 78% after adding 60% ethanol. The decrease in concentration of Fe (III) by time is shown in Figure S5b. A smaller decrease in iron (III) concentration (2.2 g/kg) is detected compared to the experiments with iron (II) (3.2 g/kg).

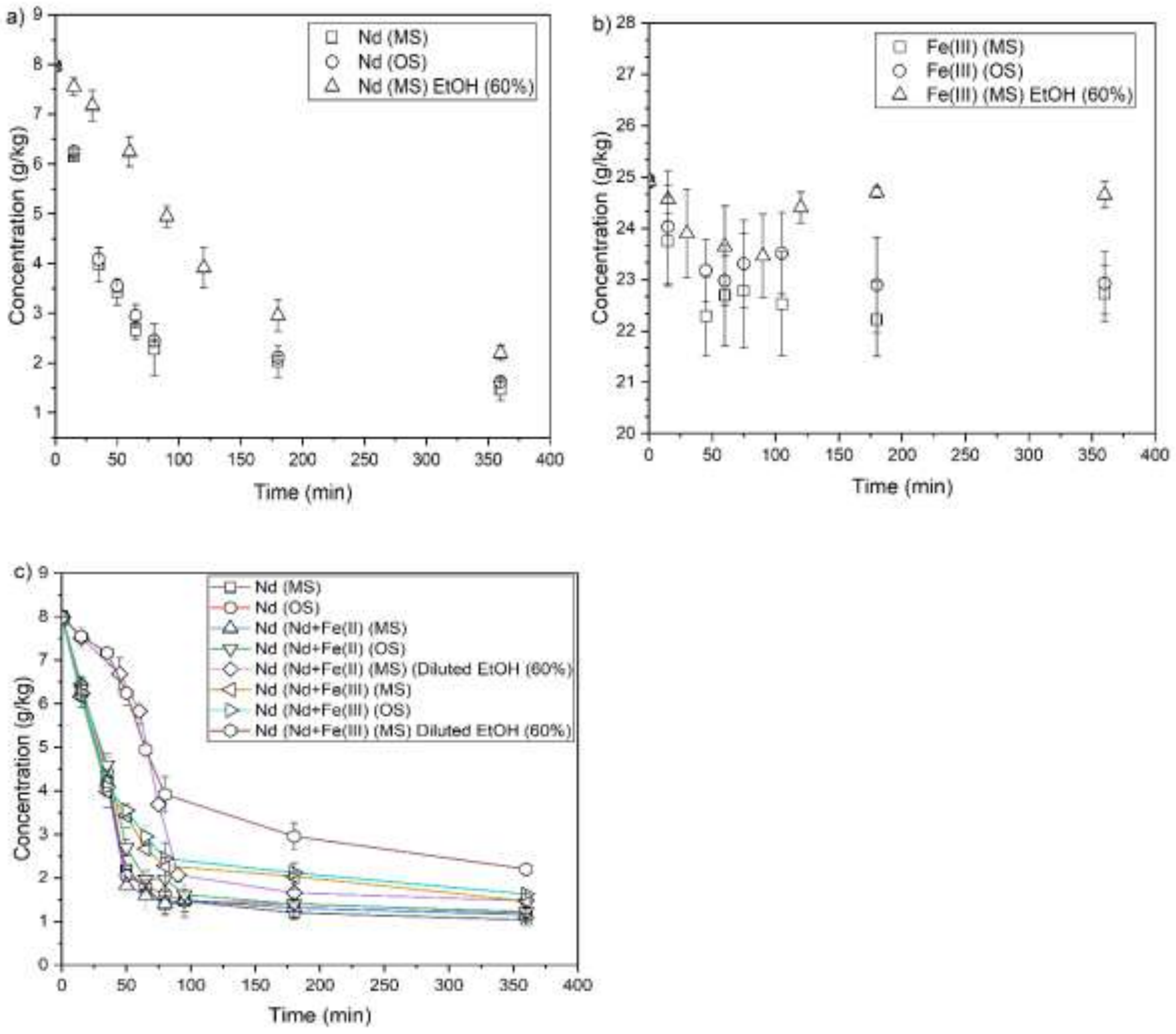

Figure S5. Desupersaturation profile for a) Nd and b) Fe II when adding (i) concentrated EtOH under magnetic stirring (MS) and overhead stirring (OS) respectively and when adding

60% EtOH under magnetic stirring (MS). c) Comparison of Nd desupersaturation profiles for Nd, Nd+Fe(II) and Nd+Fe(III) case. The antisolvent addition rate was 1 mL/min. Conc. is expressed as mass per kg initial solution.

The recovered crystals were identified as $Nd_2(SO_4)_3.8H_2O$ by powder XRD, see Figure S6.

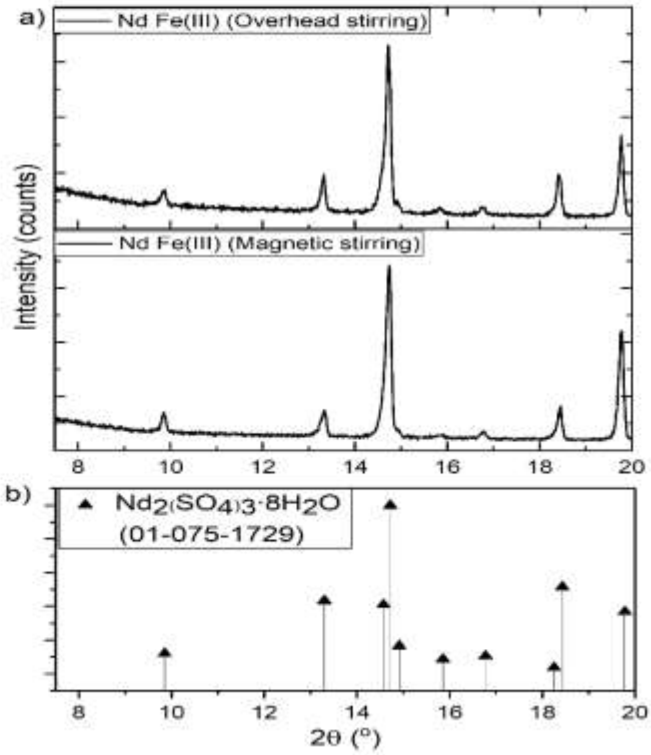

Figure S6. PXRD pattern for Nd+Fe (III) performed under magnetic and overhead stirring. Antisolvent addition rate was 1 mL/min. b) reference peaks for ($Nd_2(SO_4)_3{\cdot}8H_2O$) phases (PDF card numbers given in legends)

3.2 Seeded experiments

3.2.1 Seed characterization

The XRD patterns for Nd and Nd+Dy seeds confirming the formation of major Nd (III) sulphate octahydrate crystals ($Nd_2(SO_4)_3{\cdot}8H_2O$) phase in the crystals in both Nd and Nd+Dy seeds, see Figure S7.

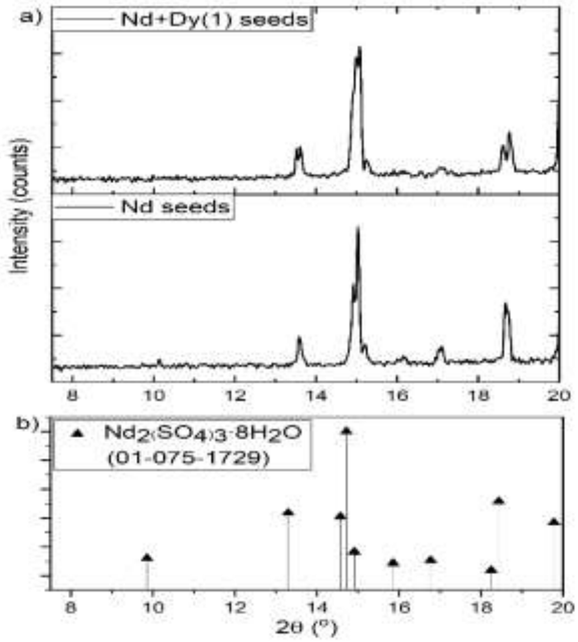

Figure S7. a) PXRD pattern of prepared Nd and Nd+Dy (1) seeds used in seeded trails. b) reference peaks for ($Nd_2(SO_4)_3{\cdot}8H_2O$) phases (PDF card numbers given in legends)

Table1. SEM-EDX analysis of seeds

Elements wt (%)	Nd seeds	Nd+Dy seeds (1)	Nd+Dy seeds (2)
Nd	39.98	49.64	44.40
Dy	n.d.	4.87	4.50
S	11.72	16.04	15.09
O	44.96	28.04	33.15

3.2.2. Seeded crystallization in presence of Fe (II) using magnetic stirring.

The decrease in neodymium concentration with time is shown in Figure S8. The neodymium concentration is consumed at a lower rate as the seed loading is increased, indicating promotion of growth over secondary nucleation. The desupersaturation profile for Fe (II) shows a small decrease in concentration over time. The decrease in Fe concentration is reduced with increased seed loading; this indicates that Fe (II) does not tend to adsorb on the seed crystals. For 10% Nd seed loading the measured iron concentration in solution is reduced by 1.5 g/kg.

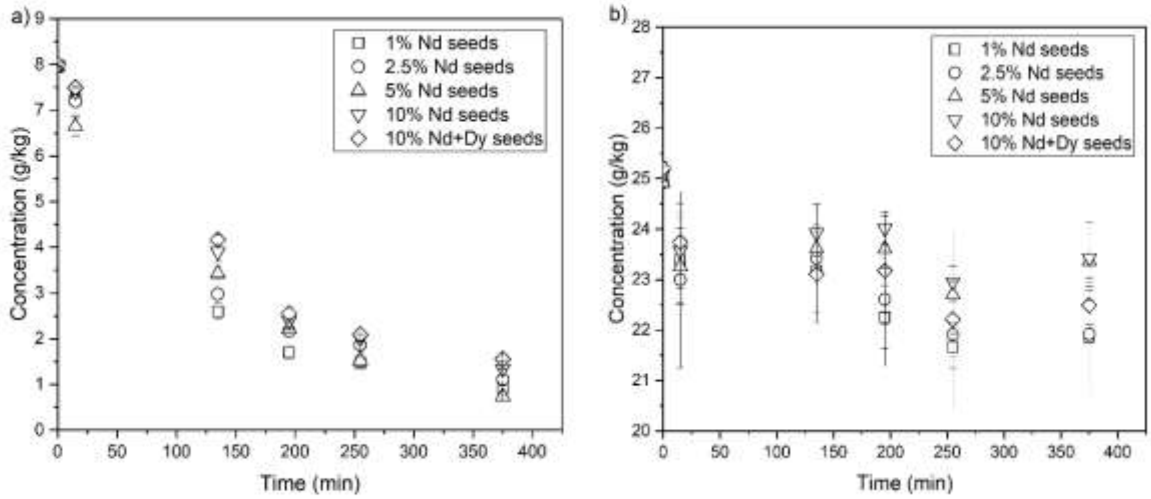

Figure S8. Desupersaturation profile for (a) Nd (b) Fe (II) with time for 1%, 2.5%, 5% 10% Nd and 10% Nd+Dy seed loading and at Fe concentration of 25 g/kg and antisolvent addition rate of 0.1 mL/min. Conc. is expressed as mass per kg initial solution.

The XRD analysis shows the formation of Nd (III) sulphate octahydrate crystals ($Nd_2(SO_4)_3.8H_2O$) in Nd and Nd+Dy seeded experiments, see Figure S9.

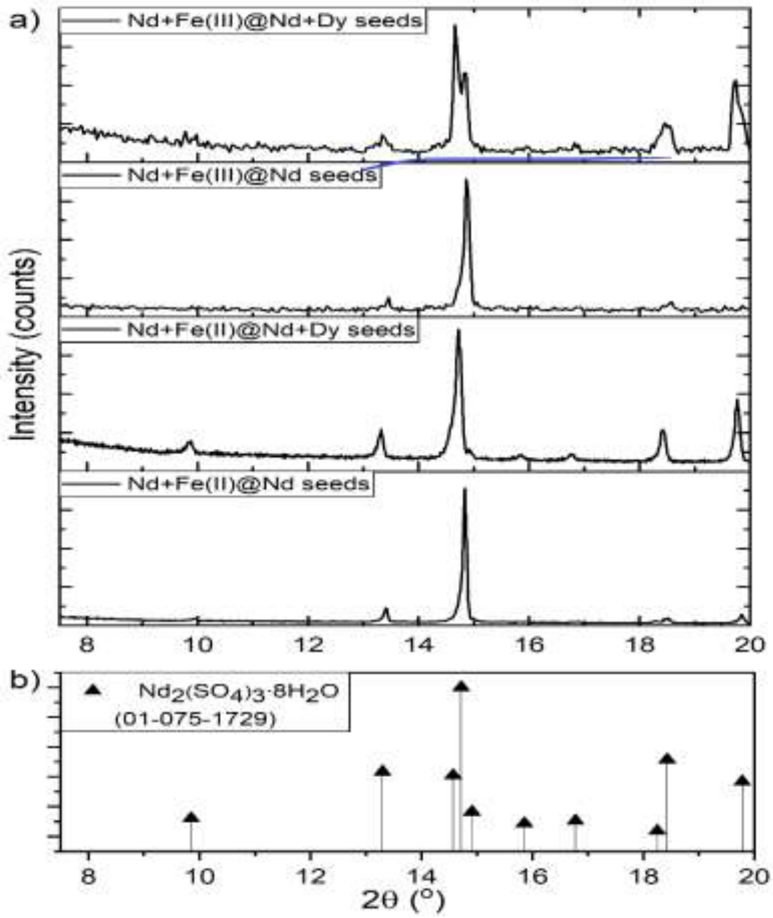

Figure S9. a) PXRD pattern of crystals recovered for 10% Nd and 10%Nd+Dy seeded trials for Fe concentration of 25 g/kg and antisolvent addition rate of 0.1 mL/min applying magnetic stirring. b) reference peaks for ($Nd_2(SO_4)_3{\cdot}8H_2O$) phases (PDF card numbers given in legends)

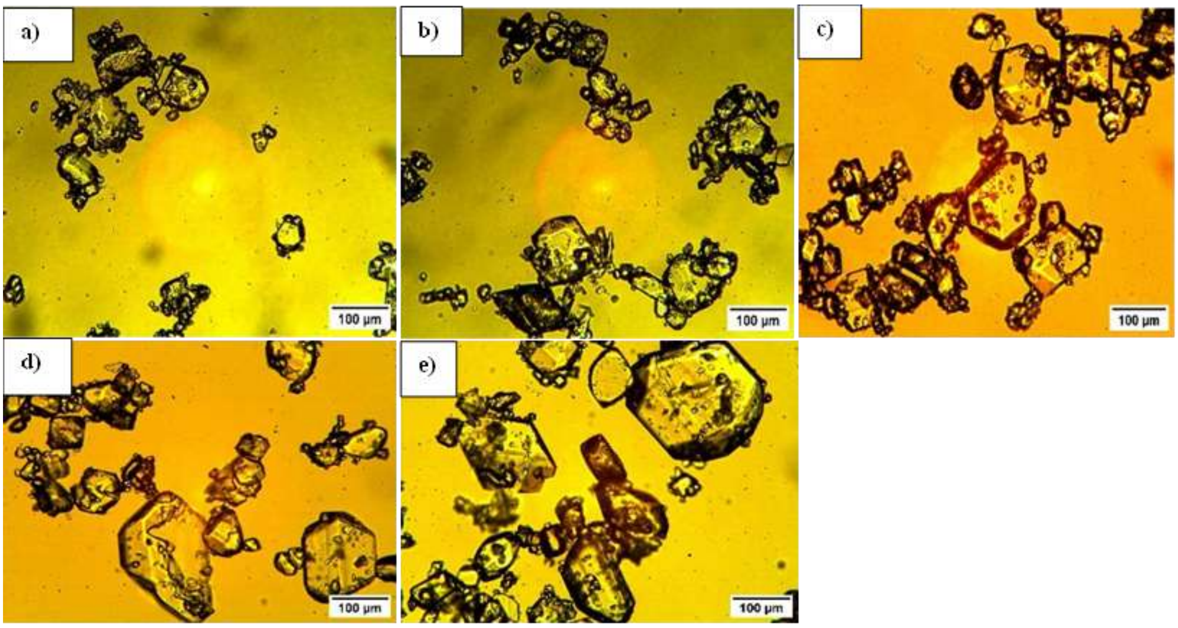

Figure S10. Micrographs of the crystals recovered for a) 1% (b) 2.5% (c) 5% and d) 10% Nd seed loading with SEM image and e) 10% Nd+Dy seed loading with corresponding SEM at Fe concentration of 25 g/kg and antisolvent addition rate of 0.1 mL/min and magnet stirring.

The crystal recovered for different seed loadings of 1%, 2.5%, 5% and 10% and an addition rate of 0.1 mL/min of concentrated ethanol to solutions initially containing 25 g/kg of Fe (II) shows hexagonal morphology (Figure S10). The size increased with seed loading with no change in morphology for the seeded trials. Seed loadings higher than 20% was not investigated further as initial experiments showed no significant change in the purity of Nd crystals. The crystals are grown much bigger in size than the original seed crystals for higher seed loading of 5 and 10%. The micrographs and scanning electron microscopy shows fewer smaller crystals for higher seed loading (5 and 10%), the smaller crystals might appear due to secondary nucleation. The seeded system with higher seed loading favoued crystal growth with reduced secondary nucleation. The secondary nucleation occurred in the system as there were not enough seed crystals present in the system to consume the supersaturation generated by dosing of antisolvent.

The CSD plot of the product crystals shows that the average crystal size obtained are 76, 88, 115, 129 µm for 1, 2.5, 5 and 10% seed loading respectively, see Figure S11. The fine crystals are neglected in this size determination. The size distribution is more narrow for lower seed loading (1 and 2.5%), and wider for higher seed loading (5 and 10%).

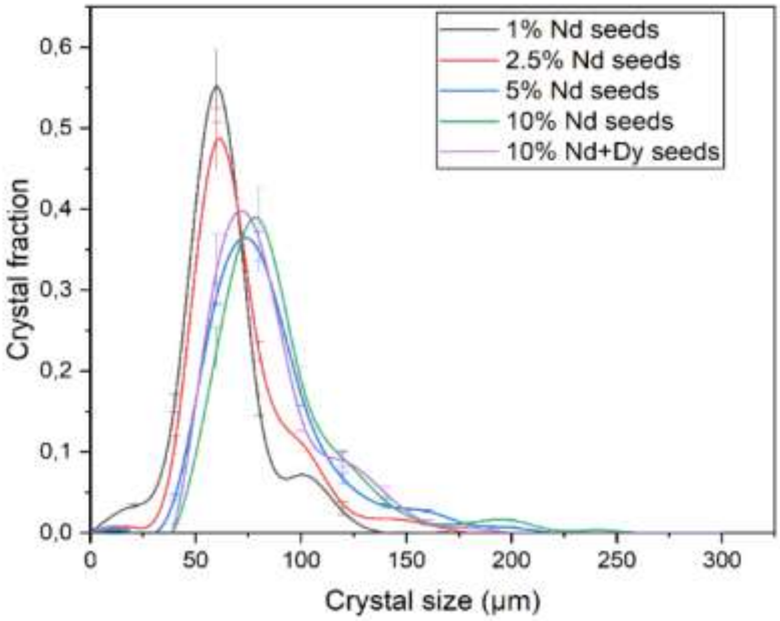

Figure S11. CSD plot of the crystals recovered for 1%, 2.5%, 5%, and 10% Nd seed loading and 10% Nd+Dy seed loading with an antisolvent addition rate of 0.1 mL/min and magnet stirring.

Experiments were also carried out at higher antisolvent addition rate i.e., 0.25 mL/min for 5 and 10% seed loading with pure Nd seeds. The antisolvent addition time for these trials was 163 min for 0.25 mL/min of addition rate. Desupersaturation profiles of neodymium and iron together with the data for the 5 and 10 % seed loading and slower addition rate (0.1 mL/min) is reported in Figure S12. The decrease in Nd is concentration faster for the higher antisolvent addition rate (0.25 mL/min) compared to the lower addition rate (0.1 mL/min). The measured decrease in Fe concentration is 2.9 and 2.7 g/kg for 5 and 10% seed loading respectively with an antisolvent addition rate of 0.25 mL/min. The decrease in Fe concentration is lower with 0.1 mL/min of addition rate, 0.57 g/kg and 1.2 g/kg for 5 and 10% seed loading respectively.

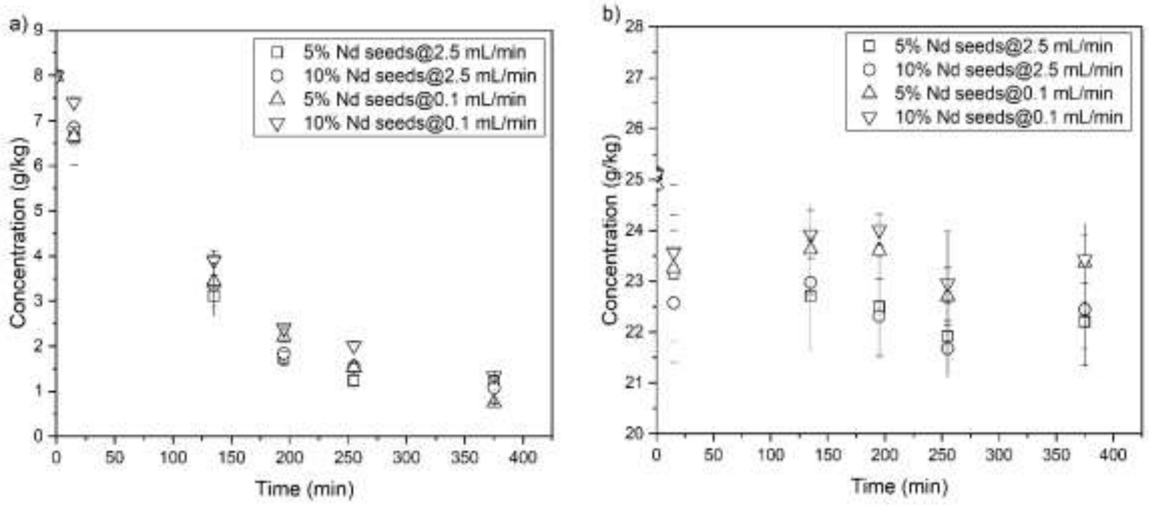

Figure S12. Desupersaturation profile for a) Nd and b) Fe (II) with time for 5% and 10% Nd seed loading and an initial Fe (II) concentration of 25 g/kg with an antisolvent addition rate of 0.25 mL/min. Conc. is expressed as mass per kg initial solution.

The micrographs show crystals of same morphology and smaller crystals at 5 and 10% seed loading for 0.25 mL/min of antisolvent addition rate (Figure S13). This could be due to possible secondary nucleation occuring in the system at higher antisolvent addition rate.

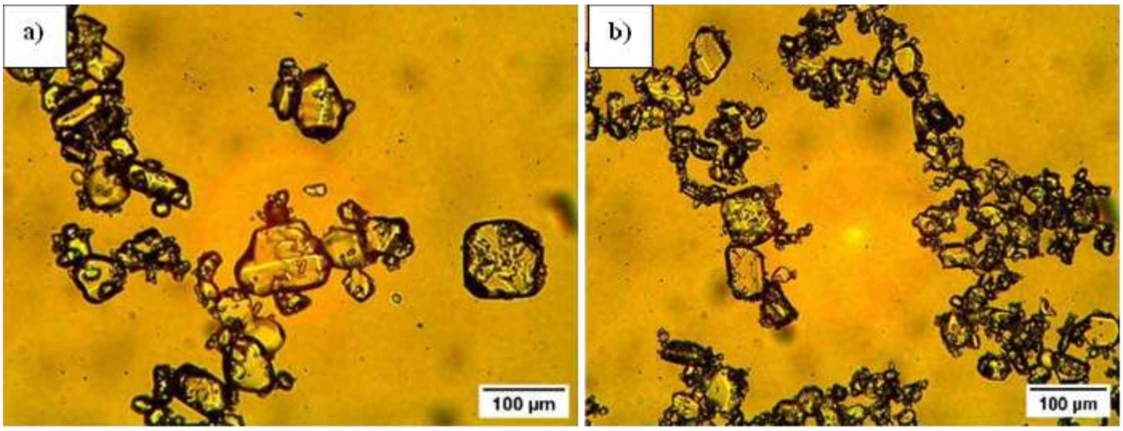

Figure S13. Micrographs of the crystals recovered for a) 5% and b) 10% seed loading and an initial Fe (II) concentration of 25 g/kg and antisolvent addition rate of 0.25 mL/min and magnet stirring.

In Figure S14, the CSD for 5 and 10 % seed loading at 0.25 mL/min of antisolvent addition rate are presented. The CSD of the crystals obtained at a lower addition rate of 0.1 mL/min and seed loading of 5% and 10% are added for comparison. The average size of crystals are 80 and 57 μm for 5 and 10 % seed loading respectively. The crystals are comparably larger

for the slower addition rate at the same seed loading, indicating promotion of growth over nucleation under these conditions.

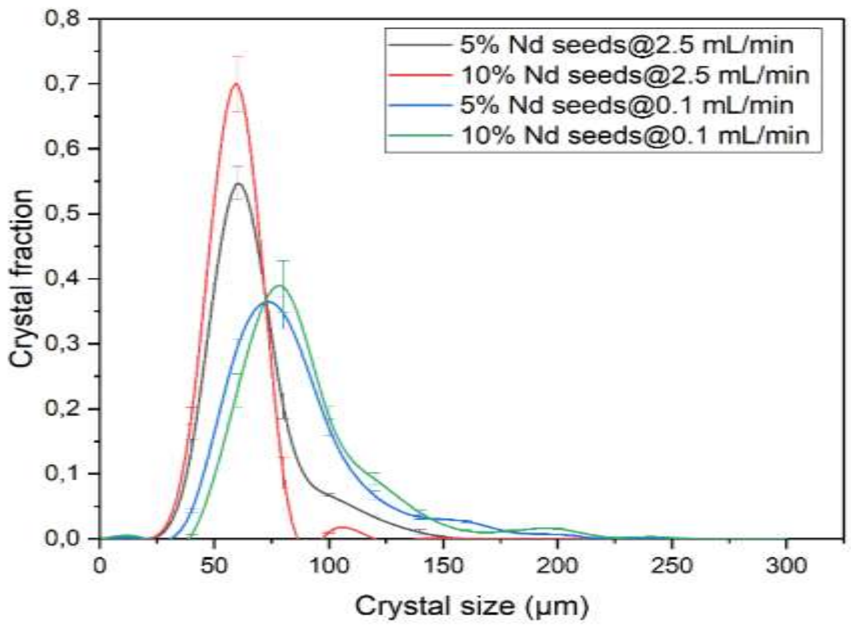

Figure S14. CSD plot of the crystals recovered for a) 5% and b) 10% Nd seed loading and at antisolvent addition rate of 0.25 mL/min and magnet stirring.

For the experiment using 10% mixed Nd and Dy seeds, the desupersaturation curve of Nd and Fe (II) is similar to the corresponding experiment using pure Nd seed crystals (Figure S9). The decrease in Fe is 1.7 g/kg for 10% Nd+Dy seed loading. The recovered crystals show similar hexagonal morphology and crystal size distribution compared to when the system was seeded with pure Nd seeds under otherwise the same conditions (Figure S10 and S11). The average crystal size obtained was 135 µm.

Experiments were also performed with lower initial Fe (II) concentration (13 g/kg) to decrease the risk for nucleation of separate iron phases. The seed loading was 10%. The change in Nd and Fe (II) concentration with time is plotted in Figure S15. The concentration of Nd decreased over time while the concentration of Fe (II) remained almost constant during the crystallization process, see Figure S15. A similar trend in concentrations was observed for both Nd and Nd+Dy seed loading.

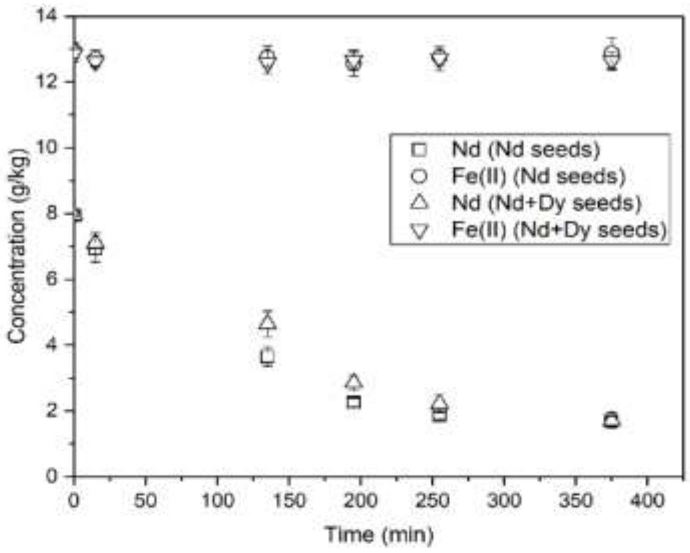

Figure S15. Desupersaturation curve for 10% Nd and 10% Nd+Dy mixed seed loading with an initial Fe (II) concentration of 13 g/kg and an antisolvent addition rate of 0.1 mL/min. Conc. is expressed as mass per kg initial solution.

The recovered crystals showed no change in morphology but resulted in smaller product crystals. The crystal size was reduced to 60 and 65 µm when using Nd and Nd+Dy seeds respectively, which is almost half of the size of crystals obtained at higher Fe (II) concentration, see Figure S16 and S17.

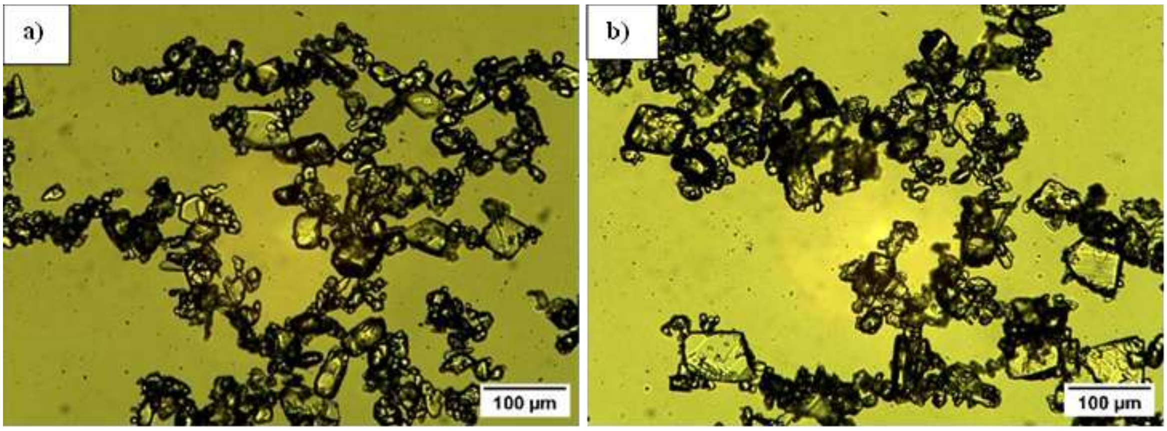

Figure S16. Micrographs of crystal recovered at Fe (II) concentration of 13 g/kg for a) 10% Nd and b) 10% Nd+Dy mixed seed loading at antisolvent addition rate of 0.1 mL/min and magnet stirring.

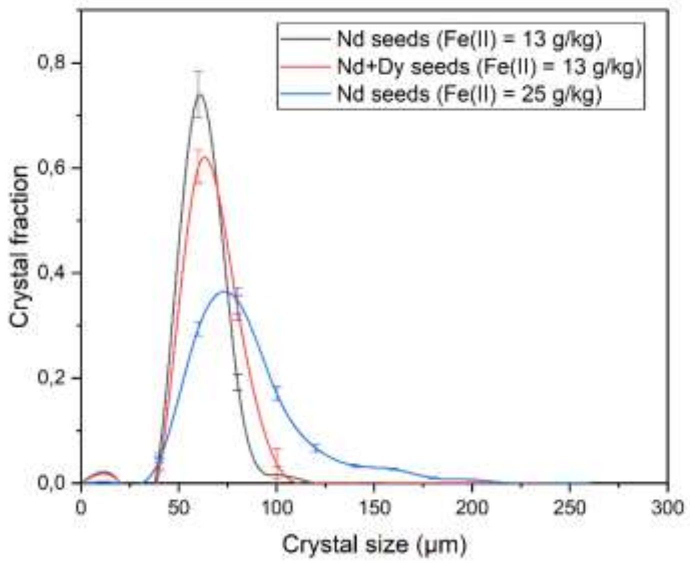

Figure S17. CSD plot for crystal recovered at lower Fe (II) concentration for a) 10% Nd and b) 10% Nd+Dy mixed seed loading at antisolvent addition rate of 0.1 mL/min and magnet stirring.

3.2.3 Seeded crystallization in presence of Fe (II) using overhead stirring.

Experiments were also performed applying overhead stirring for 10% Nd and Nd+Dy seeds (set B). The desupersaturation profiles are plotted together with magnetic stirred experiments for comparison in Figure S18. The decrease in Nd concentration is slightly higher after seeding with Nd seeds than Nd+Dy seeds. The Fe concentration showed a similar concentration profile to that observed in magnetic stirred experiments.

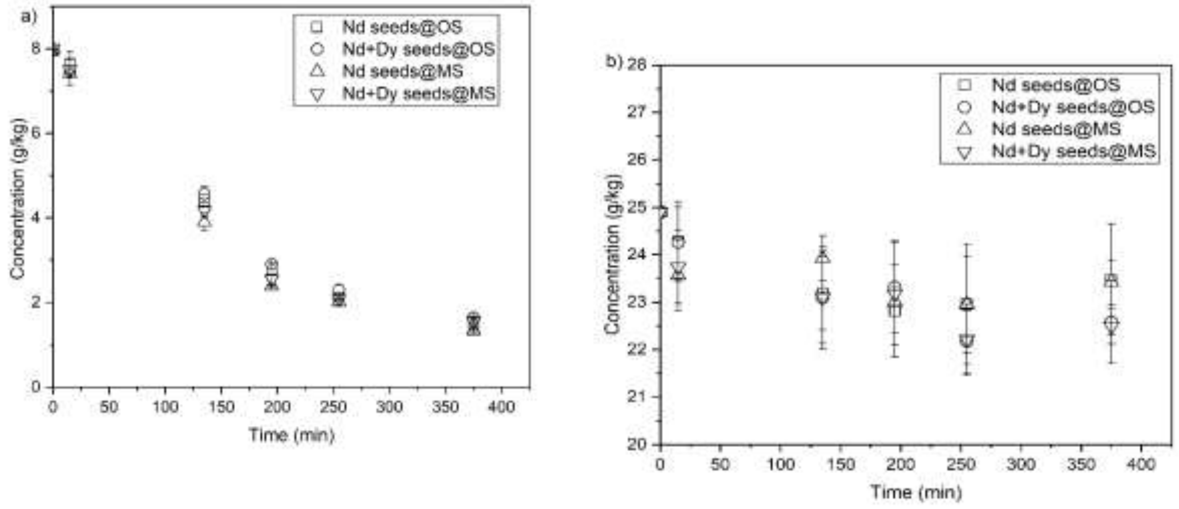

Figure S18. Desupersaturation profile for Nd and Fe (II) with time for 10% Nd and Nd+Dy seed loading at initial Fe concentration of 25 g/kg and antisolvent addition rate of 0.1 mL/min

applying overhead stirring (OS) and magnetic stirring (MS) respectively. Conc. is expressed as mass per kg initial solution.

The XRD analysis confirmed the formation of Nd (III) sulphate octahydrate crystals ($Nd_2(SO_4)_3.8H_2O$) at Fe(II) concentration of 20 g/kg for Nd and Nd/Dy seeds applying overhead stirring.

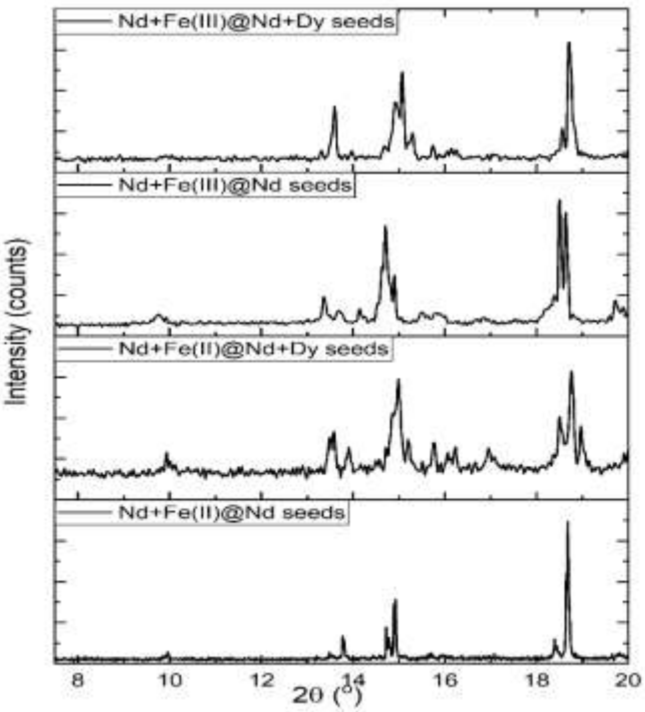

Figure S19. p-XRD patterns for crystals recovered for 10% Nd and 10% Nd+Dy seed loading at Fe concentration of 20 g/kg and antisolvent addition rate of 0.1 mL/min applying overhead stirring.

The CSD plot is shown in Figure S20. The average crystal size observed was 170 µm for pure Nd seeds and 185 µm for mixed Nd+Dy seeds. This indicates that both $Nd_2(SO_4)_3{\cdot}8H_2O$ and $(Nd/Dy)_2(SO_4)_3{\cdot}8H_2O$ act equally good as seeds for growth of $Nd_2(SO_4)_3{\cdot}8H_2O$ under these conditions.

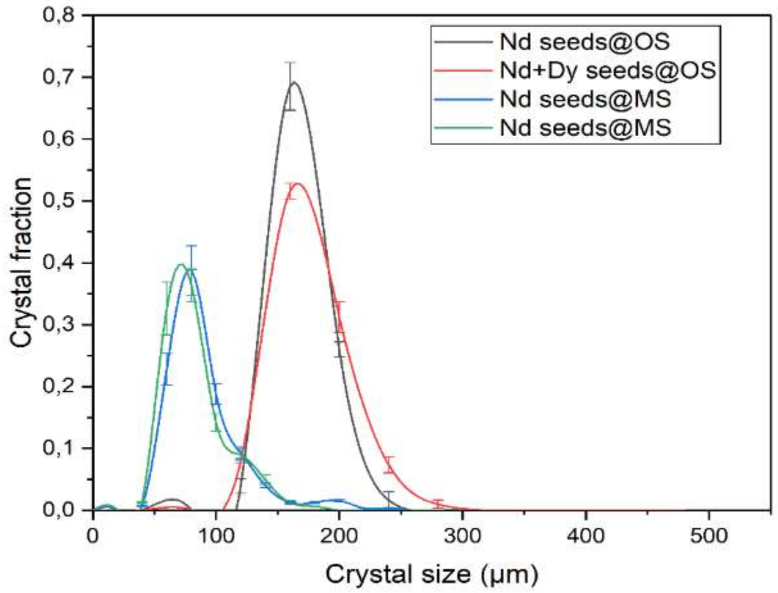

Figure S20. CSD plot for crystal recovered for a) 10% Nd seed loading and b) 10% Nd+Dy seed loading and an antisolvent addition rate of 0.1 mL/min with overhead stirring.

3.3 Seeded crystallization in presence of Fe (III)

3.3.1 Seeded crystallization in presence of Fe (III) using magnetic stirring.

Seeded crystallization was performed with a seed loading of 10% and an addition rate of 0.1 mL/min of concentrated ethanol to solutions initially containing 20 g/kg, 25 g/kg, or 13 g/kg of Fe (III). Seeds of pure Nd sulphate octahydrate and seeds of the mixed Nd+Dy phase were used.

The desupersaturation profiles show a slower decrease in Nd concentration over time compared to the unseeded experiment, see Figure S21. The decrease in Fe concentration is 1.9 and 2.1 g/kg after seeding with Nd and Nd+Dy seeds respectively, which is lower than the unseeded experiment where the decrease in Fe concentration is 2.8 g/kg.

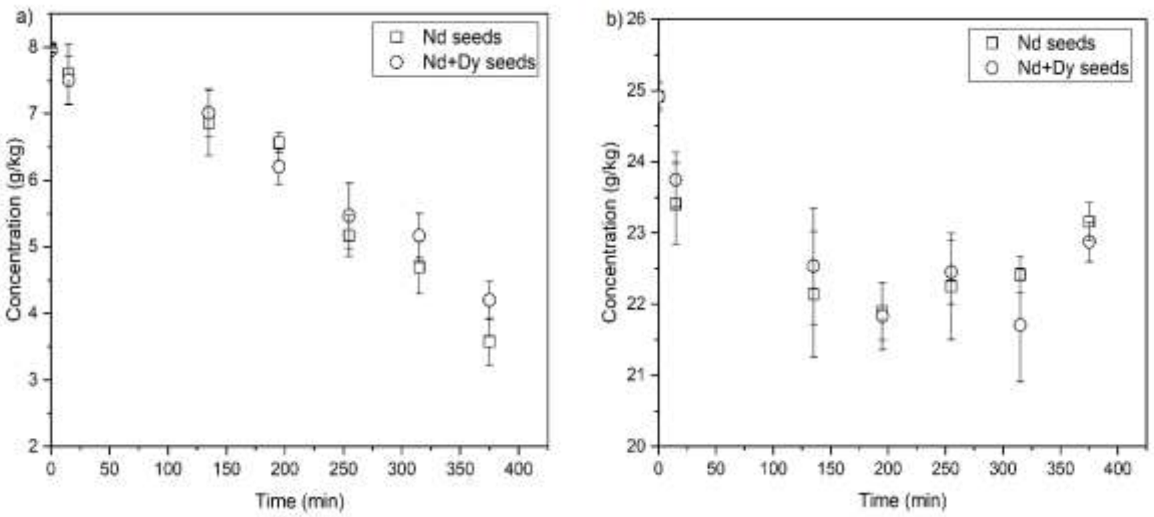

Figure S21. Desupersaturation profile for Nd and Fe (III) for 10% Nd and 10% Nd+Dy mixed seed loading at Fe concentration of 25 g/kg and antisolvent addition rate of 0.1 mL/min. Conc. is expressed as mass per kg initial solution.

The XRD analysis confirmed the formation of Nd (III) sulphate octahydrate crystals ($Nd_2(SO_4)_3{\cdot}8H_2O$) for Nd and Nd/Dy seeds applying magnetic stirring, see Figure 9. The crystals are agglomerated similar to the unseeded experiments, see Figure S22. The micrograph indicates smaller crystals surrounding the seed crystals forming agglomerates.

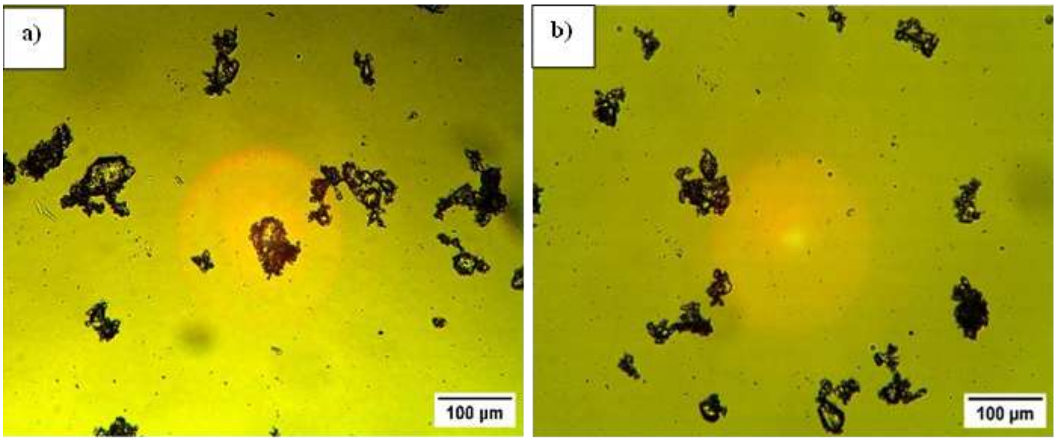

Figure S22. Micrographs of the crystals recovered a) 10% Nd and b) 10% Nd+Dy seed loading for Fe concentration of 25 g/kg and at antisolvent addition rate of 0.1 mL/min and magnetic stirring.

The decrease in Nd and Fe (III) concentration with time in the experiments conducted at the lower initial Fe (III) concentration (13 g/kg) for 10% Nd and Nd+Dy seed loading is presented in Figure S23. The desupersaturation profiles are similar for pure and mixed seeds and the Fe (III) concentration remains close to constant in the solution, see Figure S23. The

desupersaturation curves in the experiment with higher Fe (III) concentrations are plotted for comparison. The Nd concentration decreases notably faster in the experiment with lower concentration of Fe (III).

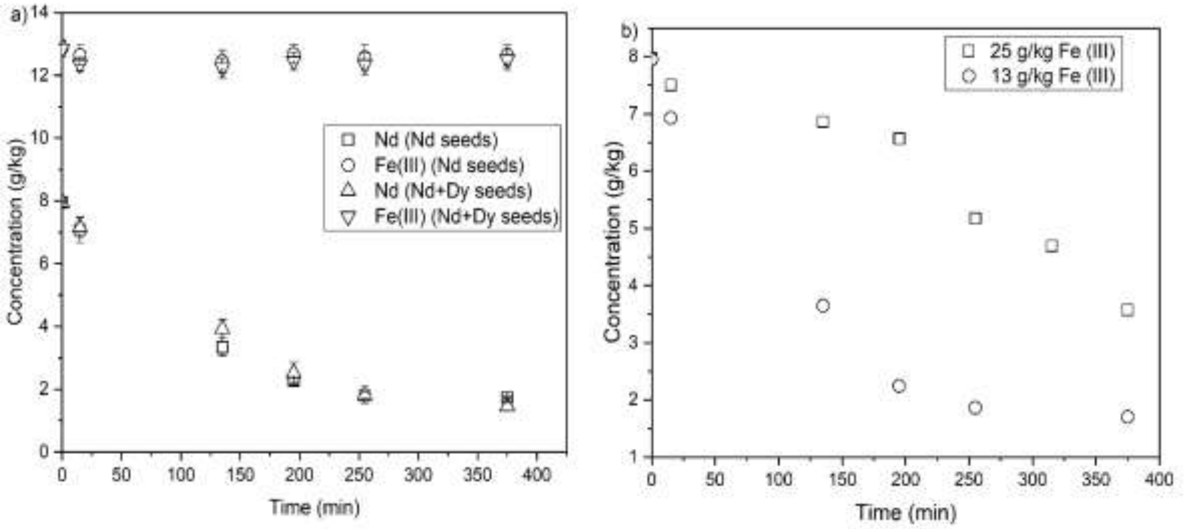

Figure S23. a) Desupersaturation curve for 10% Nd and 10% Nd+Dy mixed seed loading at Fe (III) concentration of 13 g/kg and antisolvent addition rate of 0.1 mL/min. b) Nd desupersaturation compared for 13 and 25 g/kg Fe (III). Conc. is expressed as mass per kg initial solution.

The crystals are agglomerated for both Nd and Nd+Dy seeded experiments (Figure S24) and similar to the crystals grown at the higher concentration of iron (Figure S22). The results indicate that Fe (II) has a growth inhibiting effect on $Nd_2(SO_4)_3{\cdot}8H_2O$.

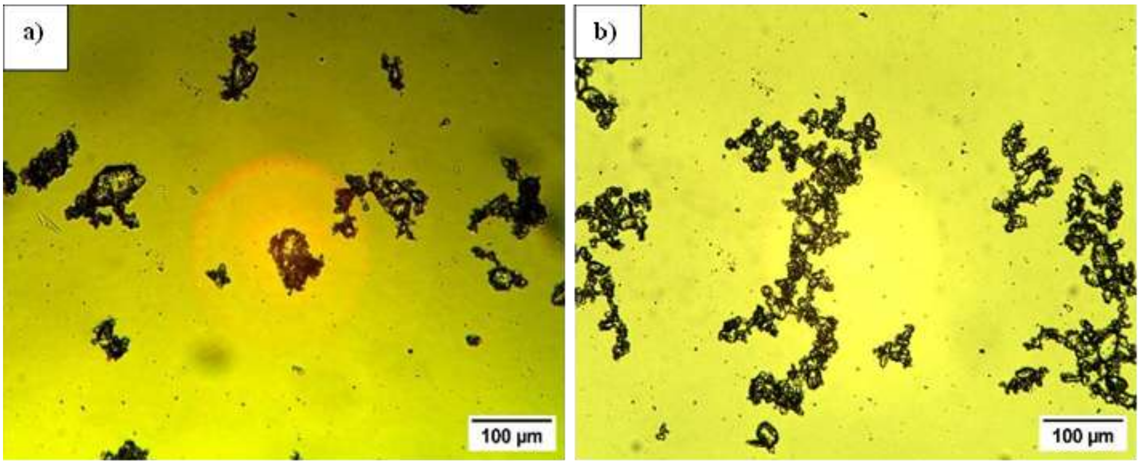

Figure S24. Micrographs of crystal recovered at Fe (III) concentration of 13 g/kg for a) 10% Nd and b) 10% Nd+Dy mixed seed loading at antisolvent addition rate of 0.1 mL/min.

3.3.2 Seeded crystallization in presence of Fe (III) under overhead stirring.

Experiments were performed for an initial Fe concentration of 25 g/kg utilizing overhead stirring for 10% Nd and Nd+Dy seeds (set B). The desupersaturation profile is plotted together with magnetic stirred experiments for comparison, see Figure S25. The desupersaturation curves of Nd is similar in magnetic and overhead stirred experiments. The Fe concentration showed a similar concentration profile as observed in magnetic stirred experiments.

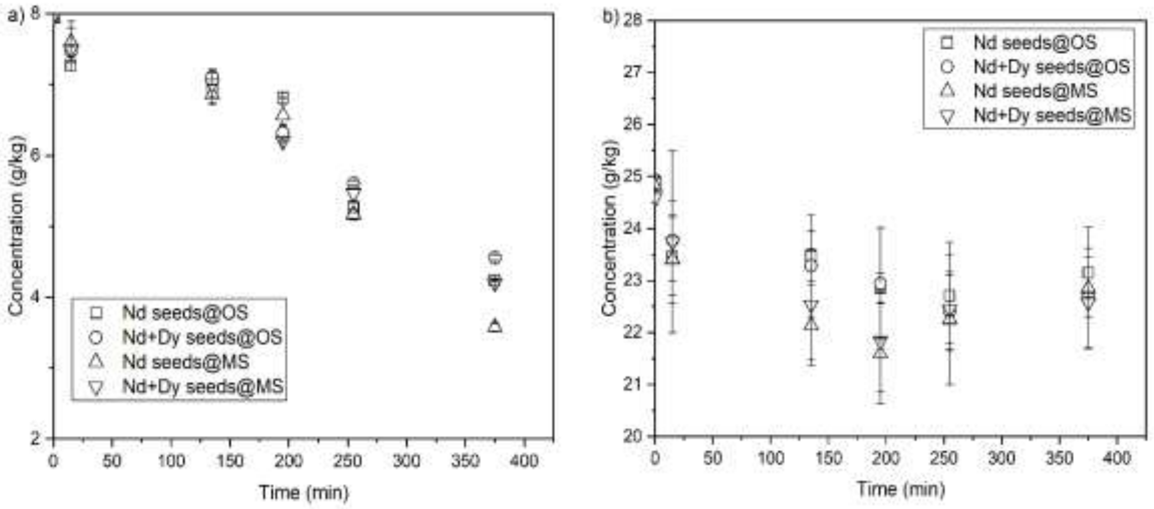

Figure S25. Desupersaturation profile for Nd and Fe (III) at 10% Nd and 10% Nd+Dy mixed seed loading for Fe concentration of 25 g/kg at antisolvent addition rate of 0.1 mL/min carried out under overhead stirring. Conc. is expressed as mass per kg initial solution.

The XRD analysis confirmed the formation of Nd (III) sulphate octahydrate crystals ($Nd_2(SO_4)_3.8H_2O$) at Fe(III) concentration of 20 g/kg for Nd and Nd/Dy seeds applying overhead stirring, see Figure S19.

3.5 Solid phase purity

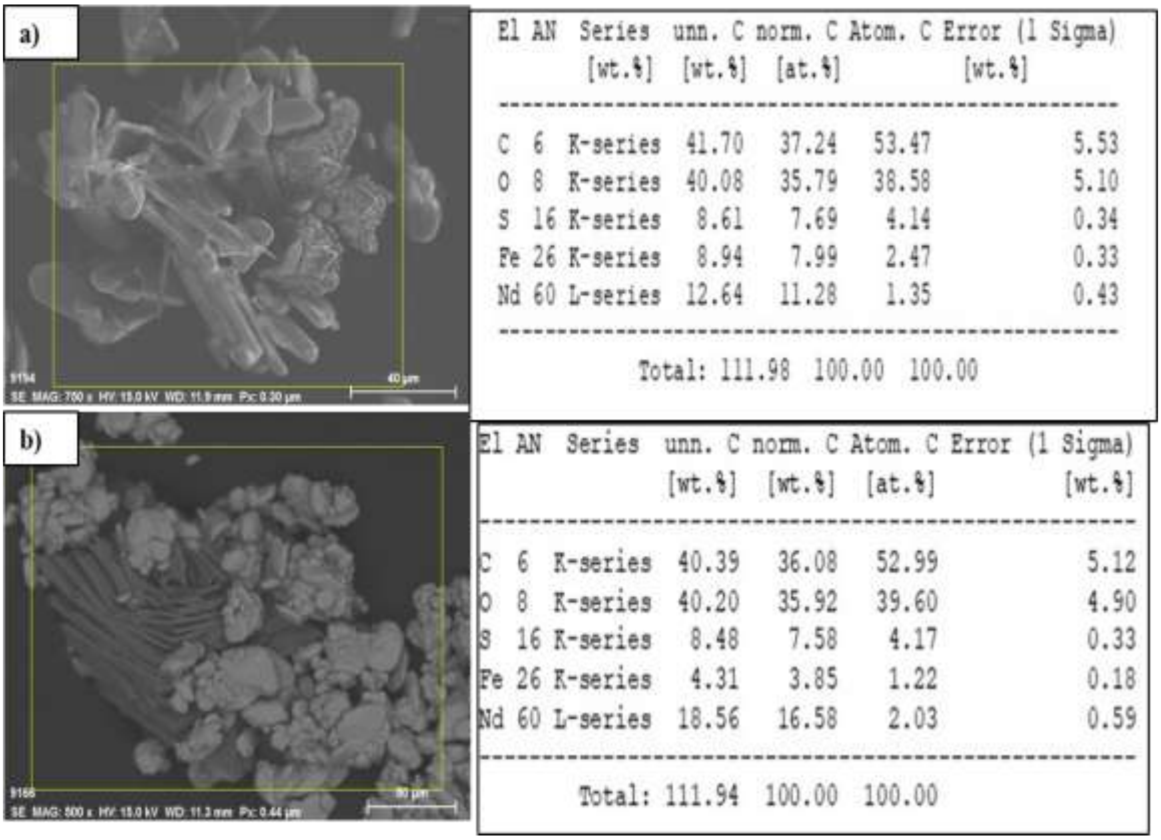

a)

El	AN	Series	unn. C [wt.%]	norm. C [wt.%]	Atom. C [at.%]	Error (1 Sigma) [wt.%]
C	6	K-series	41.70	37.24	53.47	5.53
O	8	K-series	40.08	35.79	38.58	5.10
S	16	K-series	8.61	7.69	4.14	0.34
Fe	26	K-series	8.94	7.99	2.47	0.33
Nd	60	L-series	12.64	11.28	1.35	0.43
		Total:	111.98	100.00	100.00	

b)

El	AN	Series	unn. C [wt.%]	norm. C [wt.%]	Atom. C [at.%]	Error (1 Sigma) [wt.%]
C	6	K-series	40.39	36.08	52.99	5.12
O	8	K-series	40.20	35.92	39.60	4.90
S	16	K-series	8.48	7.58	4.17	0.33
Fe	26	K-series	4.31	3.85	1.22	0.18
Nd	60	L-series	18.56	16.58	2.03	0.59
		Total:	111.94	100.00	100.00	

Figure S26. The surface elemental composition of a) Fe (II) and b) Fe(III) detected with EDX mapping for Fe concentration of 25 g/kg at antisolvent addition rate of 1 mL/min carried out under magnetic stirring.

3.5.1 Statistical significance of Purity

For purity, where only small differences were observed, statistical significance was assessed using a one-way ANOVA. Purity measurements obtained under 14 different experimental conditions of Fe(II) and Fe(III), each with 3 replicate measurements (Count = 3), are used to test whether the variation in purity is statistically significant. The p-value obtained was 0.0059, which is well below the 0.05 threshold ($p < 0.05$), whereas F calculated is higher than F critical indicating that the differences are statistically significant.

Anova: Single Factor							
SUMMARY							
Trial	*Seed type*	*Seed loading*	*Stirring*	*Count*	*Sum*	*Average*	*Variance*
Nd+Fe(II)	Nd seeds	10%	Magnetic	3	298.2	99.4	0.07
Nd+Fe(II)	Nd+Dy seeds	10%	Magnetic	3	297.3	99.1	0.07
Nd+Fe(II)	Nd seeds	10%	Overhead	3	298.5	99.5	0.06

Nd+Fe(II)	Nd+Dy seeds	10%	Overhead	3	297.6	99.2	0.06
Nd+Fe(II)	Nd seeds	1%	Magnetic	3	292.8	97.6	0.20
Nd+Fe(II)	Nd seeds	2.5%	Magnetic	3	293.4	97.8	0.23
Nd+Fe(II)	Nd seeds	5%	Magnetic	3	296.7	98.9	0.25
Nd+Fe(II)	Nd seeds	5%	Magnetic	3	290.1	96.7	0.71
Nd+Fe(III)	Nd seeds	10%	Magnetic	3	297.6	99.2	0.27
Nd+Fe(III)	Nd seeds	10%	Overhead	3	297.6	99.2	0.27
Nd+Fe(III)	Nd+Dy seeds	10%	Magnetic	3	297.6	99.2	0.25
Nd+Fe(III)	Nd+Dy seeds	10%	Overhead	3	296.7	98.9	0.23
Nd+Fe(III)	-	-	Magnetic	3	294.9	98.3	0.22
Nd+Fe(III)	-	-	Overhead	3	294.6	98.2	0.25

**ANOVA**						
Source of Variation	*SS*	*df*	*MS*	*F cal*	*P-value*	*F crit*
Between Groups	10.60	13	0.81	3.09	0.0059	2.08
Within Groups	7.41	28	0.26			
Total	18.01	41				
